# Investigating diversity and similarity between CBM13 modules and ricin-B lectin domains using sequence similarity networks

**DOI:** 10.1186/s12864-024-10554-1

**Published:** 2024-06-27

**Authors:** Tibo De Coninck, Garry P. Gippert, Bernard Henrissat, Tom Desmet, Els J.M. Van Damme

**Affiliations:** 1 Laboratory of Biochemistry and Glycobiology, Department of Biotechnology, Ghent University, Proeftuinstraat 86, Ghent, 9000 Belgium; 2 Section for Protein Chemistry and Enzyme Technology, Department of Biotechnology & Biomedicine, Technical University of Denmark, Søltofts Plads 224, Kgs. Lyngby, 2800 Denmark; 3 Centre for Synthetic Biology, Department of Biotechnology, Ghent University, Coupure Links 653, Ghent, 9000 Belgium

**Keywords:** CAZymes, Carbohydrate-binding modules, ricin-B lectins, Chimerolectins

## Abstract

**Background:**

The CBM13 family comprises carbohydrate-binding modules that occur mainly in enzymes and in several ricin-B lectins. The ricin-B lectin domain resembles the CBM13 module to a large extent. Historically, ricin-B lectins and CBM13 proteins were considered completely distinct, despite their structural and functional similarities.

**Results:**

In this data mining study, we investigate structural and functional similarities of these intertwined protein groups. Because of the high structural and functional similarities, and differences in nomenclature usage in several databases, confusion can arise. First, we demonstrate how public protein databases use different nomenclature systems to describe CBM13 modules and putative ricin-B lectin domains. We suggest the introduction of a novel CBM13 domain identifier, as well as the extension of CAZy cross-references in UniProt to guard the distinction between CAZy and non-CAZy entries in public databases. Since similar problems may occur with other lectin families and CBM families, we suggest the introduction of novel CBM InterPro domain identifiers to all existing CBM families. Second, we investigated phylogenetic, nomenclatural and structural similarities between putative ricin-B lectin domains and CBM13 modules, making use of sequence similarity networks. We concluded that the ricin-B/CBM13 superfamily may be larger than initially thought and that several putative ricin-B lectin domains may display CAZyme functionalities, although biochemical proof remains to be delivered.

**Conclusions:**

Ricin-B lectin domains and CBM13 modules are associated groups of proteins whose database semantics are currently biased towards ricin-B lectins. Revision of the CAZy cross-reference in UniProt and introduction of a dedicated CBM13 domain identifier in InterPro may resolve this issue. In addition, our analyses show that several proteins with putative ricin-B lectin domains show very strong structural similarity to CBM13 modules. Therefore ricin-B lectin domains and CBM13 modules could be considered distant members of a larger ricin-B/CBM13 superfamily.

**Supplementary Information:**

The online version contains supplementary material available at 10.1186/s12864-024-10554-1.

## Background

Carbohydrate-active enzymes (CAZymes) constitute a highly diverse group of biocatalysts involved in the breakdown, synthesis and modification of oligosaccharides, polysaccharides and glycoconjugates. In the CAZy database, a distinction is made between glycoside hydrolases (GH), glycosyl transferases (GT), polysaccharide lyases (PL), carbohydrate esterases (CE), modules with auxiliary activities (AA) and carbohydrate-binding modules (CBM) [1]. CBMs do not exert enzymatic activity but are typically present in multi-domain proteins in combination with a catalytic domain [2, 3]. These CBMs are classified in families and clans based on amino acid similarity and typical conserved protein fold. Furthermore, they can also be classified according to their carbohydrate-binding specificity, where type A, B and C CBMs show affinity for crystalline polysaccharides, glycans and small sugars, respectively [4]. This study is focussed on the CBM13 family.

Lectins are another group of proteins that show carbohydrate-recognition and -binding properties [5]. By definition, lectins are carbohydrate-binding proteins that lack enzymatic activity [6, 7]. Similar to CBMs, the group of lectins can be classified in several families based on their amino acid sequences. One of these families is the ricin-B lectin family, for which ricin, a phytotoxin from castor bean (*Ricinus communis*) is the founding member. Ricin is built up of two chains, the A (*a*ctive) and B (*b*inding) polypeptides are linked by a disulphide bridge [8]. The A-chain displays toxicity because of its N-glycosidase activity (EC 3.2.2.22) towards rRNA or DNA, resulting in the release of adenine residues. Originally, ribosomes were considered as the main target of the A-chain, hence the nickname of ‘ribosome-inactivating protein’ (RIP) was given to proteins containing the A-chain [9, 10]. The B-chain of ricin is composed of two ricin-B lectin domains and exhibits carbohydrate-binding properties, most often towards galactose, lactose and/or N-acetylgalactosamine. These two ricin-B lectin domains are considered the result of gene duplication events [8, 11]. Each ricin-B lectin domain is composed of four β-strands, arranged into a β-trefoil with threefold pseudo-symmetry [12]. At amino acid sequence level, each ricin-B lectin domain consists of three homologous repeats (α, β and γ) of around 50 amino acids, each of which contains at least one conserved QXW-motif and two cysteine residues [13]. All modules classified in the CBM13 family basically show the same characteristics as those of the ricin-B lectin domain [14]. In addition, these modules can also be observed in various CAZymes [15–19], next to their prevalence in a wide variety of lectins from plants, fungi and animals [20–31].

Lectin domains and CBMs have been considered as completely distinct protein domains for a very long time. Nevertheless, several lectins consisting only of carbohydrate-binding domain(s) and lectin domains as part of multi-domain proteins have been classified in multiple CBM families. Moreover, it was shown that several type-C CBMs and lectins interact with carbohydrates in a thermodynamically indistinguishable manner [4]. Several years ago, it was reported that many plant lectin sequences encode chimeric proteins composed of a lectin domain, in combination with ‘unrelated’ non-lectin domains, such as a protein kinase domain, F-box domain or GH domain similar to CAZymes [32, 33]. The observation that some lectins can occur in a chimeric domain architecture in combination with a CAZyme domain, opposes the ancient paradigm that lectins do not exhibit catalytic activity.

**Fig. 1:**
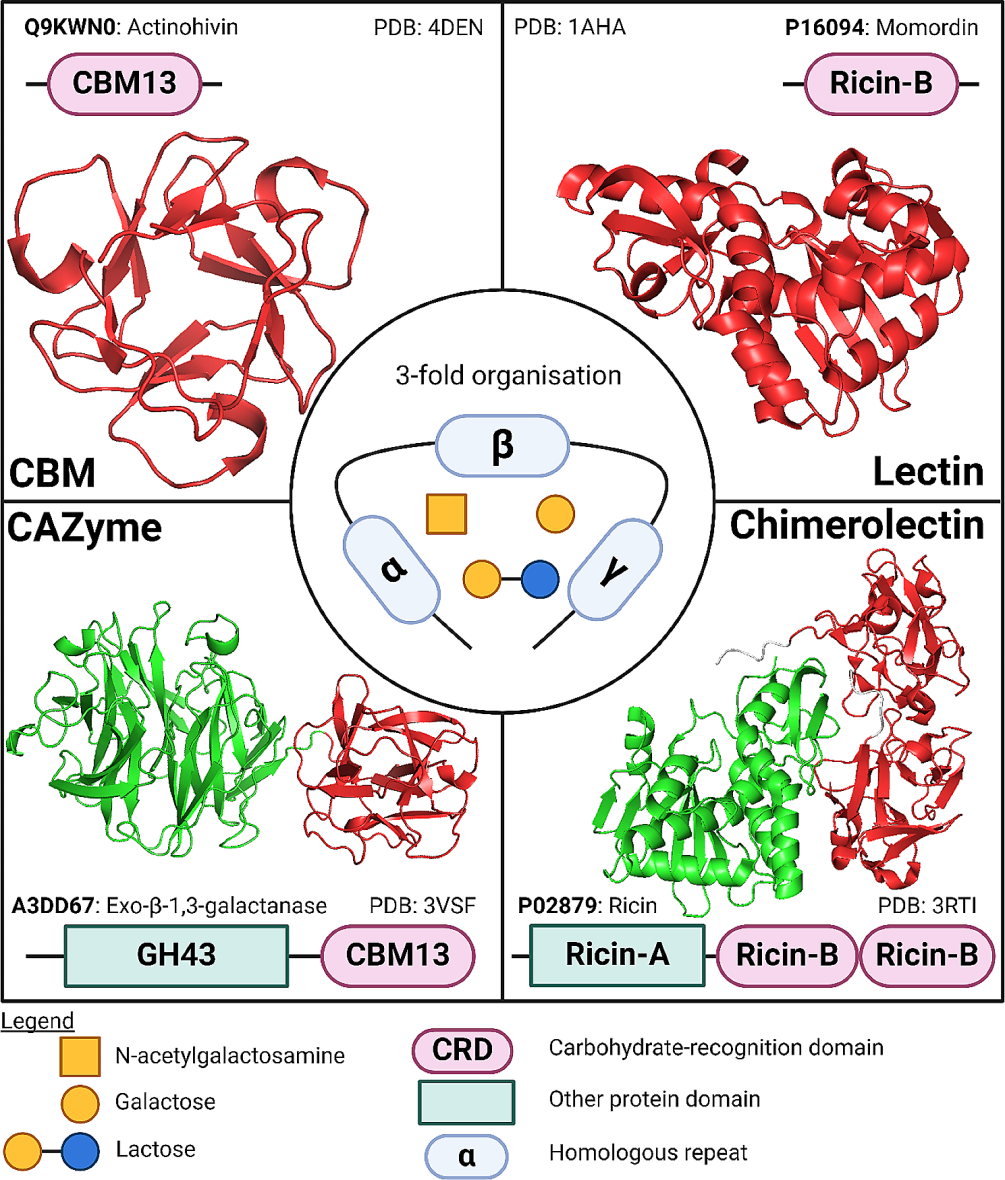
Visual comparison of CBMs, CAZymes, lectins and chimerolectins. Domain architectures and corresponding three-dimensional structures of examples of a monomeric CBM13, CBM13-containing CAZyme, a ricin-B lectin and a chimeric ricin-B lectin are depicted. Carbohydrate-recognition domains are shown in red, while other protein domains are shown in green. Typical homologous repeats of the CBM13/ricin-B carbohydrate-recognition domain are shown in pale blue and denominated as α, β and γ. CBMs are usually part of a larger CAZyme but can sporadically occur as single-module proteins. Lectins contain at least one lectin domain and can occur as single-domain proteins or in a chimeric configuration with other non-lectin domains. Typically, CBM13 modules and ricin-B lectin domains can bind reversibly to N-acetylgalactosamine, galactose and/or lactose. UniProt entry codes of the example proteins are given in the figure. Protein structures were obtained from www.release.rcsb.org/ and edited in PyMol

The boundaries between lectins and CBMs become even thinner when structural resemblances are considered, since several CBMs found on CAZymes show structural similarity towards particular lectins. For example, the CBM6-containing GH11-xylanase from *Clostridium stercorarium* is structurally very similar to the fucose-binding fucolectin from *Anguilla Anguilla* [34]. Interestingly, there are also examples of proteins consisting of only a single CBM, without any other protein domain, including a malectin (CBM57) from *Xenopus laevis* and tachytin (CBM14) from *Tachypleus tridentatus* [35, 36]. The observation that CBMs can occur as single-domain proteins, grants lectin properties to certain CBMs, and demonstrates how vague the distinction between lectins and CBMs is in some cases (Fig. 1).

Nowadays, experiments focussing on altering the carbohydrate-binding affinity of lectins are being executed by combining multiple CBMs [37]. These ‘neolectins’ are created for a diverse array of research and diagnostic applications within the glycobiology field [38]. This exemplifies how the world of lectins and CBMs are overlapping with each other.

To study similarities between proteins, traditionally phylogenetic trees are used. However, with the complete sequencing of genomes, the amount of available biological information has increased explosively. Consequently, the demand for new methods to handle these large datasets has increased likewise [39, 40]. Sequence similarity networks (SSN) are used to analyse and visualise relationships between biological sequences and have been proven useful to investigate the sequence-structure-function relationships in large datasets in a timely and biologically meaningful manner [41–44]. The use of SSNs is widely accepted and is being incorporated as a tool to investigate taxonomical, phylogenetical, structural and functional characteristics of proteins, CAZymes and other enzymes [45–48].

The aim of this study is to demonstrate the complex semantical differences and similarities between ricin-B lectin domains and CBM13 modules, as well as to show the phylogenetical, taxonomical, functional and structural diversity amongst these groups of proteins.

## Materials and methods

### The ricin-B/CBM13 sequence space and nomenclature analysis

**Fig. 2:**
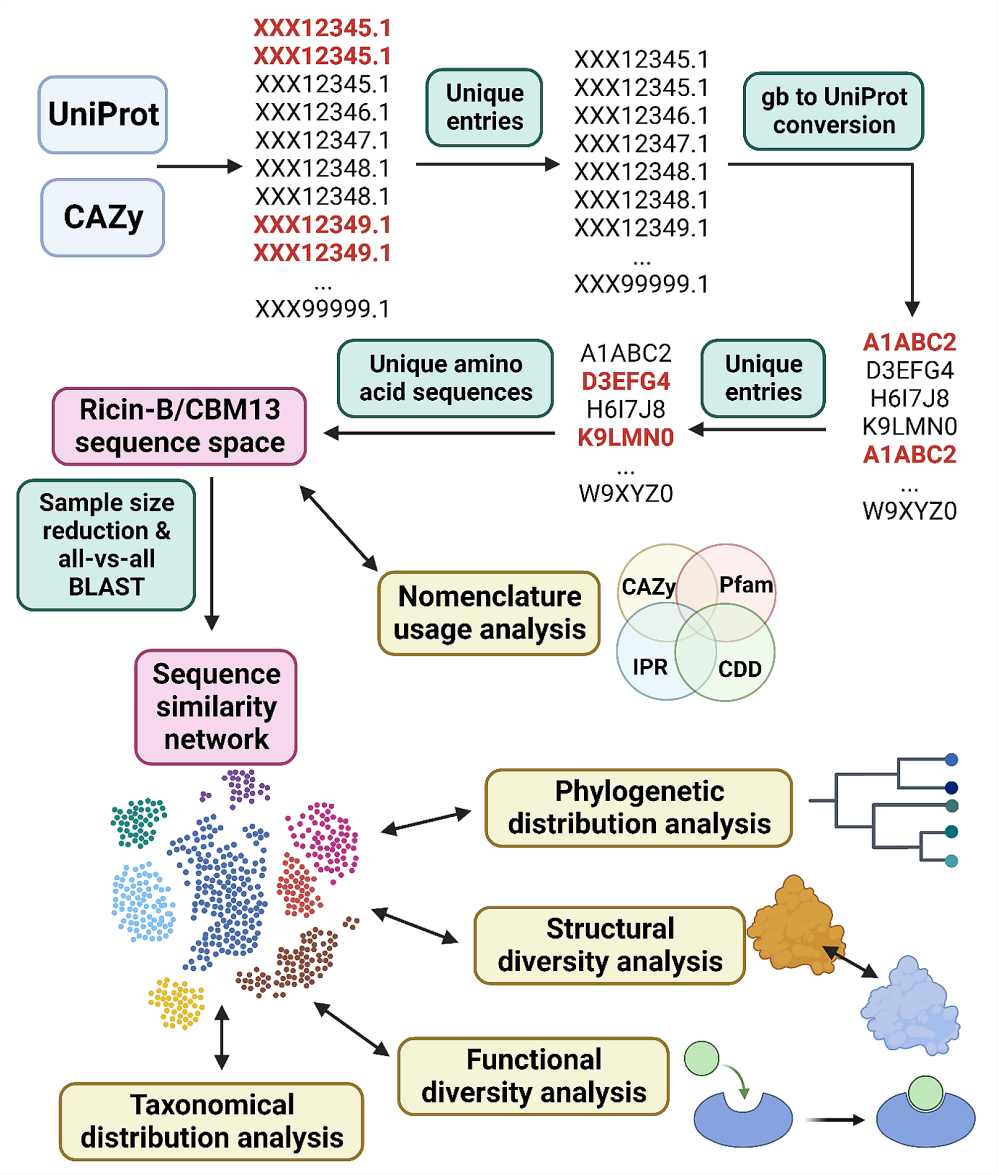
Creation of the ricin-B/CBM13 sequence space and sequence similarity network. Ricin-B and CBM13-related entries were searched for in UniProt and CAZy. In UniProt, relevant entries were retrieved using ‘ricin’ and ‘CBM13’ as keywords. In CAZy, Genbank entries were directly downloaded from the dedicated CBM13 webpage. Multiple selection and interconversion steps were employed to obtain a final set of unique amino acid sequences. The ricin-B/CBM13 sequence space is used to create sequence similarity networks through sample size reduction and all-vs-all BLAST. The final sequence similarity network is used for taxonomical distribution analysis, functional diversity analysis, structural diversity analysis and phylogenetic distribution analysis

The ricin-B/CBM13 sequence space, encompassing all sequences associated with ricin-B lectins and CBM13 proteins, was created by combining UniProt [49, 50] and the CAZy database [1, 51] as two main sources of sequences (Fig. 2). In UniProt, ricin-B/CBM13-related protein entries were searched using “ricin” and “CBM13” as keywords. CBM13 entries were available via the CAZy database. Sequences were downloaded from NCBI [52]. Metadata (i.e. protein names, Gene Ontology (GO) annotations, protein domain identifiers) were obtained through UniProt. Two major criteria were enforced when compiling the sequence space: 1) every entry should be represented in both Genbank and UniProt; and 2): every entry must have a unique amino acid sequence. To meet these criteria, a multi-step entry conversion procedure was employed (Fig. 2), considering: (1) multiple Genbank entries can correspond to the same UniProt entry [53]; (2) several Genbank entries are not connected to a UniProt entry; (3) multiple UniProt entries can have identical amino acid sequences [54]. Therefore, the initial set of Genbank entries was screened for unique entries, converted to UniProt entries and filtered for unique amino acid sequences.

The consistency of nomenclature usage by protein databases was investigated since each database integrated in UniProt utilises different systems to classify and annotate protein domains [55]. Relevant domain identifiers were retrieved by screening the ricin-B/CBM13 sequence space for re-occurring identifiers. Entries annotated with a domain identifier from database *x* belong to the eponymous subspace *x*. Since entries occur in multiple databases, they belong to multiple subspaces. Overlap between subspaces in the use of domain nomenclature was investigated by means of Venn diagrams [56].

### Creation of sequence similarity networks

A database consisting of all CBM13 modules (available as of April 3, 2023) was compiled. Amino acid sequences for the complete ricin-B/CBM13 sequence space were submitted to BLASTp [57] against the compiled CBM13 database. Calculations were performed on the local DTU-HPC cluster [58] and a standard BLAST threshold significance value of E = 10^− 5^ was employed. BLASTp alignments were sorted by significance and identity and filtered by module length. Per NCBI query, only the single best alignment to the pool of compiled CBM13 modules was retained.

To reduce the necessary computing power and time, the set of aligned modules was reduced fivefold. First, the modules were ranked by decreasing prediction quality (i.e. increasing E-value and decreasing count of identical aligned residue positions). Then, every fifth module was retained, giving rise to five possible sets, i.e. each one containing 1/5 of the initially predicted modules. This study was performed with the first set of fivefold-reduced modules.

The SSN was generated by submitting the reduced module set (size *n*) to all-versus-all BLASTp, yielding a list of pairwise-aligned modules (network file), with *n*∙(*n* + 1)/2 pairwise module comparisons, attributed with pairwise alignment scores (BLAST score) and significance values (E-value). Modules present in any SSN are called ‘nodes’ and are connected to other nodes through ‘edges’, according to a chosen threshold E-value. Thus, E-values are used to reduce the SSN to contain only pair-wise sequence alignments with an E-value below a selected significance threshold level. The list of remaining nodes is referred to as ‘nodes list’. The network file is considered the skeleton of the SSN onto which biological metadata can be projected to enrich and combine the phylogenetical analyses with biochemically/biologically relevant characteristics [44], to gain insight in structural and functional diversity. Relevant metadata includes: taxonomy information, CAZy membership, length of (predicted) CBM13 modules, number of QX[F; W;Y] modules, BLAST scores and protein existence (PE) levels.

### Functional and taxonomical composition of the ricin-B/CBM13 SSN

The overall ricin-B/CBM13 SSN is divided into a ‘CBM13’ and a ‘putative ricin-B lectin’ subdivision. The distribution of protein and enzyme activities was investigated by inventorying the GO terms and protein names occurring in both SSN subdivisions. Protein names were categorised as ‘CAZyme’, ‘lectin related’, ‘other enzyme activities’ or ‘other’ if proteins did not fall under one of the first three categories mentioned. GO terms as of UniProt release 2023_03 were used.

### Phylogenetical and structural diversity of the ricin-B/CBM13 SSN

Within the SSN, five clusters from different taxonomical origins, with at least one CBM13 member, were selected and isolated. Module sequences were extracted and investigated by means of the phylogeny.fr pipeline, utilizing the MUSCLE algorithm for multiple sequence alignment combined with Gblocks curation and the maximum likelihood algorithm in the PhyML phylogeny program [59]. Phylogenetic trees were formatted in the FigTree v1.4.4 [60] and inkscape v1.3.2 [61] software. Within the five selected clusters, AlphaFold models of one CBM13 module and two CBM13-predicted ricin-B lectin modules were submitted to domain superimposition using the *cealign* algorithm in PyMol v2.5.4 [62, 63]. Structural alignment was evaluated based on root-mean square deviation (RMSD) values [64]. RMSD values below 2.00 Å were considered as good alignments [65]. Additionally, sequence alignments between CBM13 modules and CBM13-predicted ricin-B lectin modules were executed in ClustalOmega to obtain identity scores [66]. Multiple sequence alignments were used as input for the WebLogo webserver to study sequence conservation [67].

### Visualisations and statistical analyses

Venn diagrams were created using an online Venn diagram generator [56] and reformatted in the Inkscape software v1.3.2 [61]. Bar charts and histograms were generated and visualised by means of Microsoft Excel [68]. SSNs were visualised in the Cytoscape software [41]. Other diagrams were created in BioRender [69].

Goodness-of-fit calculations were executed using the *F*-distributed Chi-squared (${X^2}$) test with *ν* degrees of freedom (df). Correlations were calculated by means of Pearson’s r. The Welch’s t-test for samples with unequal variances with *ν* df was used to compare averages. Throughout this study, significance levels at *p* < 0.05 were enforced. All statistical calculations were performed in SPSS [70].

## Results and discussion

### The ricin-B/CBM13 sequence space

**Fig. 3:**
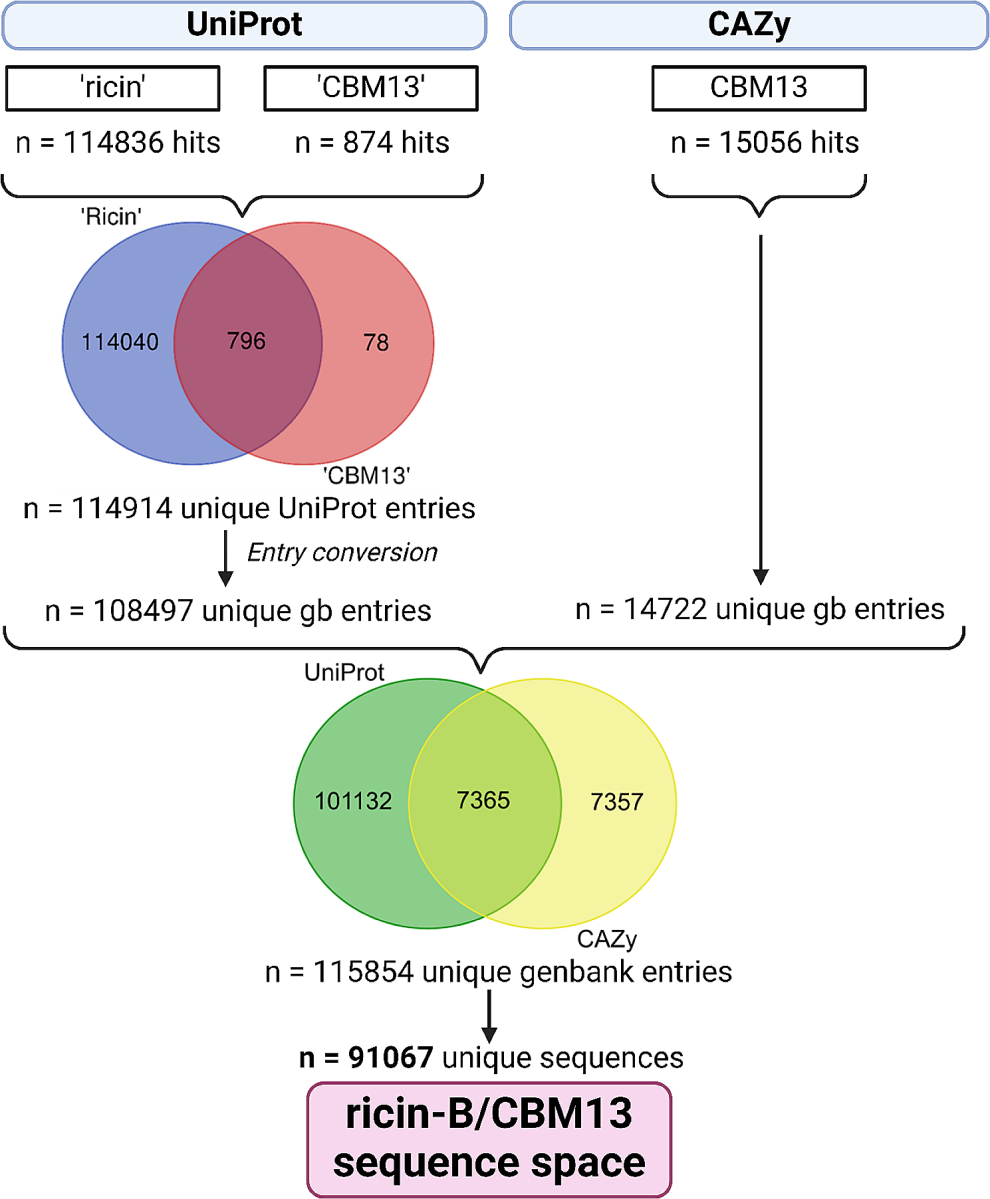
Overview of the filtering strategy to obtain the ricin-B/CBM13 sequence space. The UniProt and CAZy database were used to extract ricin-B/CBM13 entries. UniProt entries were converted to Genbank entries, combined with Genbank entries from the CAZy database and filtered for unique amino acid sequences, yielding the final ricin-B/CBM13 sequence space of 91,067 entries

A total of *n* = 14,722 Genbank entries were extracted from the CBM13 webpage, representing the set of official CBM13 members. The use of ‘ricin’ or ‘CBM13’ as keywords in UniProt yielded *n* = 114,914 entries, with the majority being retrieved also by using ‘ricin’ as keyword (Fig. 3). Finally, we obtained a set of *n* = 91,067 entries with unique amino acid sequences (Supplementary File S1, Supplementary File S2 Supplementary File S3).

During the compilation process, issues of identical sequences and redundant metadata were identified and pruned from the sequence/metadata cohorts. On average, every unique UniProt entry corresponded to 1.75 Genbank entries. The discrepancy between the number of unique UniProt entries and unique Genbank entries is a known problem (i.e. reference/accession multiplicity) and originates from redundancy within Genbank [53].

### Proteins with putative ricin-B lectin domains and CBM13 modules are indistinguishable based on nomenclature usage

**Fig. 4:**
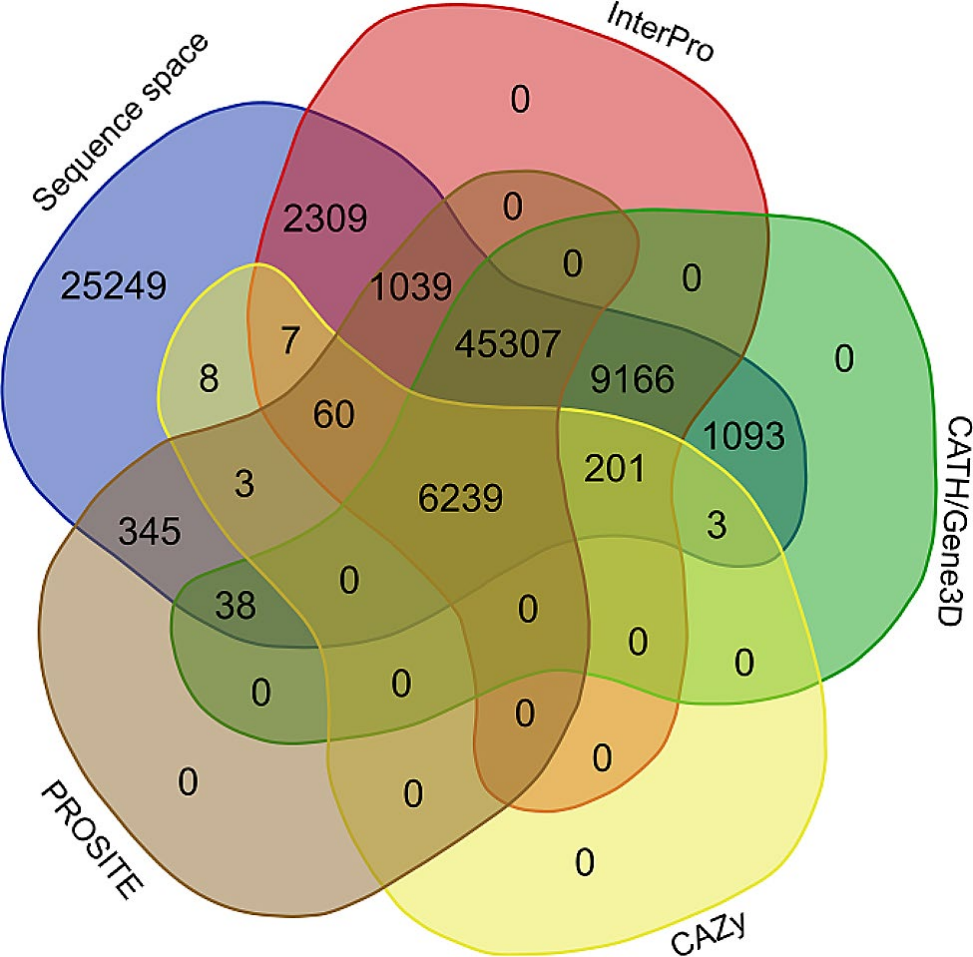
Venn diagram depicting the number of entries with overlapping domain identifiers from public databases. Each coloured lobe represents either the complete ricin-B/CBM13 sequence space (blue) or a subset thereof: the InterPro subspace (red), the CATH/Gene3D subspace (green), the CAZy subspace (yellow) or the PROSITE subspace (brown). A total of *n* = 65,818 entries (72.3% of sequence space) belongs to any of the aforementioned subspaces, while *n* = 25,249 entries (27.7%) are not attributed with any protein domain identifier. A total of *n* = 6239 entries is attributed with protein domain identifiers from all aforementioned subspaces

Identifiers describing ricin-B lectin and/or CBM13-related domains and modules were retrieved by screening the ricin-B/CBM13 sequence space for re-occurring identifiers and sorting the identifiers by prevalence (Table 1). The highest degree of sequence space coverage by identifiers was obtained by combining identifiers from InterPro, CATH/Gene3D and PROSITE (Fig. 4). InterPro identifiers covered the highest percentage of all ricin-B/CBM13-related entries. The remaining entries could not be annotated by any other relevant identifier. Since consideration of CDD, Pfam, SUPFAM and/or SMART identifiers did not increase the coverage of the sequence space, they were considered as redundant and not taken into account.

**Table 1:** Ricin-B and CBM13 proteins can be described by several protein domain identifiers

Database	Identifier	Identifier name	Overlapping entries	Sequence space coverage (%)
InterPro	IPR000772 IPR035992 IPR040249	Ricin B, lectin domain Ricin B-like lectins Ricin B-like lectin EULS3-like	54,953 63,939 737	60.3 70.2 0.8
	*Total InterPro*		64,328	70.6
CATH/Gene3D	2.80.10.50	CATH Superfamily 2.80.10.50	62,047	68.1
PROSITE	PS50231	Lectin domain of ricin B chain profile	53,031	58.2
CDD	cd00161	Ricin	50,363	55.3
Pfam	PF00652 PF14200	Ricin-type beta-trefoil lectin domain Ricin-type beta trefoil lectin domain-like	32,420 17,827	35.6 19.6
	*Total Pfam*		49,961	54.9
SMART	SM00458	Ricin-type beta-trefoil	39,166	43.0
SUPFAM	SSF50370	Ricin B-like lectins	63,462	69.7
CAZy	CBM13	Carbohydrate Binding Module family 13	6,521	7.2
UniProt	CAZy-CBM13	CAZy cross-reference in UniProt	635	0.7
Total mapped entries			65,818	72.3
Not mapped entries			25,249	27.7
Total unique sequences in the ricin-B/CBM13 sequence space			91,067	100.0

Public databases make use of specific nomenclature to describe protein domains. Per ricin-B/CBM13-related protein domain identifier, it is shown how many entries are attributed with particular domain identifiers as well as the relative coverage of the ricin-B/CBM13 sequence space by the domain identifier. Most of the sequence space is attributed with InterPro or SUPFAM identifiers. The combination of InterPro, CATH/Gene3D and PROSITE gave rise to the highest coverage rate of 72.3%

Only 7% of the total ricin-B/CBM13 sequence space comprised of CBM13 entries. The remaining 93% is occupied by putative ricin-B lectins (Table 1). The largest share of mapped protein domain identifiers originated from InterPro, of which IPR000772 and IPR035992 are the main representatives. Conversely, the IPR040249 identifier also bears ‘Ricin-B’ in its name, but is associated with *Euonymus europaeus* lectins, which are not related to ricin-B lectins [71]. The IPR040249 identifier is associated to ricin, is because the *E. europaeus* lectins also contain QXW lectin motifs [13].

**Fig. 5:**
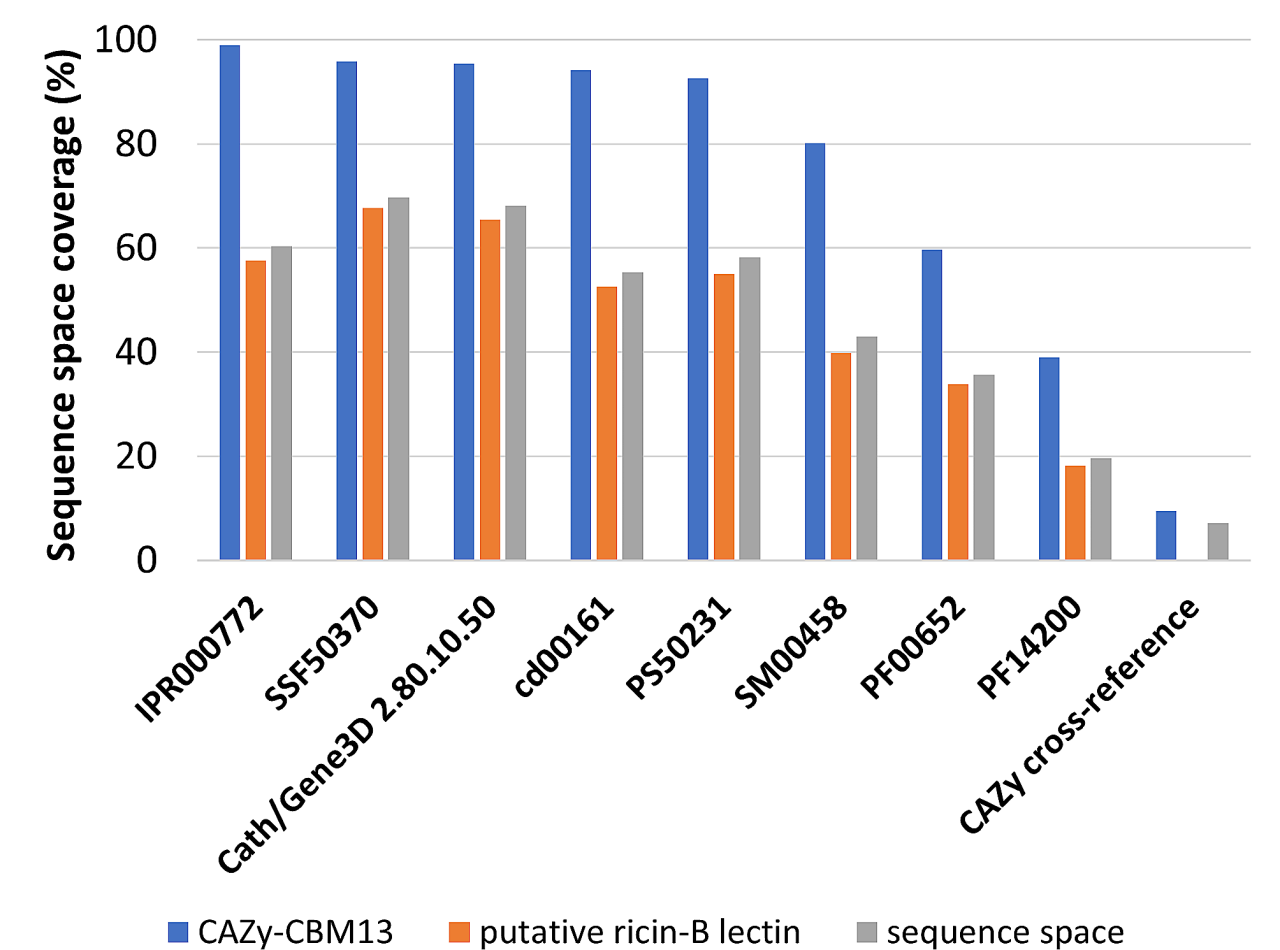
Ricin-B/CBM13-related identifier coverage for CBM13 and putative ricin-B lectins. CBM13 entries are significantly more covered by ricin-B/CBM13 domain identifiers compared to putative ricin-B lectins

The nomenclature to describe protein domains within the ricin-B/CBM13 sequence space is explicitly associated with ricin, because of the strong historical association between CBM13 and ricin-B lectins, as discussed earlier. Indeed, it appears that both groups of proteins utilise the same ricin-B related semantics (Table 2). In other words, there is currently no dedicated CBM13 identifier in UniProt or InterPro. Although both groups utilise the same identifiers, they differ significantly in the coverage rate by each domain identifier (Fig. 5). CBM13 proteins are most extensively described by identifiers from public databases. The majority (> 80%) of the CBM13 entries is described by at least InterPro, CATH/Gene3D, PROSITE, CDD, SUPFAM and SMART. Putative ricin-B lectin entries show significantly less coverage by protein domain identifiers ($\text{?}$ = 7779.027; $\text{?}$ = 1 df; one-sided *p* < 0.001). Therefore, the degree of coverage could be utilised to discriminate between CBM13 and putative ricin-B lectin entries. However, a cut-off value or threshold should be considered.

**Fig. 6:**
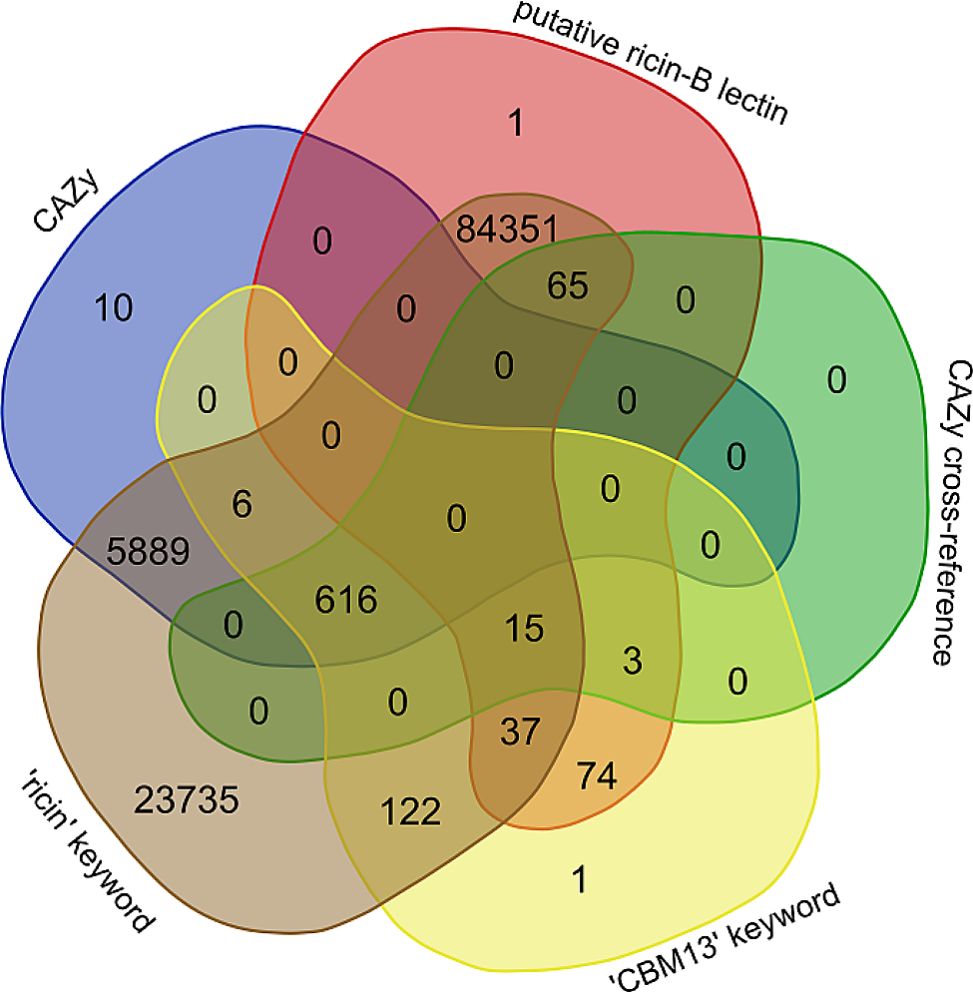
Venn diagram showing the relationship between CAZy membership, keyword usage and CAZy cross-reference in UniProt. Most of the ricin-B/CBM13-related entries are obtained by using ‘ricin’ as keyword in UniProt rather than ‘CBM13’. Only a minority of the CBM13 entries are attributed with the CAZy cross-reference in UniProt.

Nowadays, UniProt contributes to distinguishing putative ricin-B lectins from CBM13 entries by providing a dedicated CAZy cross-reference. Entries with this cross-reference should be considered as official CBM13 entries. However, in the UniProt database, membership to CBM13 is only highlighted to a lesser extent, since only 9.5% (*n* = 616) of the CBM13 entries refer to CAZy (Fig. 5). A similar observation is true for the larger collection of CAZy entries in UniProt [1]. Consequently, the majority (90.5%) of the CBM13 entries are considered as false negatives in this analysis, although they are proven CAZy members. Surprisingly, also a number of false positives (*n* = 83) were identified (i.e. putative ricin-B lectins with CAZy cross-reference), including several GHs (*n* = 41), GTs (*n* = 10), PLs (*n* = 2) and CAZymes with other CBM modules (*n* = 11) (Fig. 6) (Supplementary File S4). Similarly, when the number of hits in UniProt obtained by using ‘ricin’ or ‘CBM13’ as keywords is compared to the number of CBM13 entries, it becomes clear that 90% of the CBM13 entries is retrieved by using ‘ricin’ as a keyword rather than ‘CBM13’. The ‘CBM13’ keyword also delivers several CBM13 (*n* = 6) and putative ricin-B lectin (*n* = 129) entries without CAZy cross-reference (Fig. 6).

**Table 2:** Overview of representative CBM13 and putative ricin-B lectin entries

Protein name	Category	CAZy member	Organism	Genbank ID	UniProt ID	Domain coordinates (InterPro)	IPR035992	IPR000772	CATH/Gene 3D 2.80.10.50	PROSITE PS50231
Exo-β-1,3-galactanase	CAZyme	Yes	*Acetovibrio thermocellus* ATCC 27,405	ABN51896.1	A3DD67	353–491	X	X	X	X
β-L-arabinopyranosidase 27 A	CAZyme	Yes	*Streptomyces avermitilis* MA 4680	BAC69897.1	Q82L26	530–658	X	X	X	X
Xylanase 10 A	CAZyme	Yes	*Streptomyces lividans* 1326/IAF18	AAC26525.1	P26514	353–477	X	X	X	X
UDP-α-N-acetylgalactosaminyltransferase 9	CAZyme	Yes	*Drosophila melanogaster*	AAF57964.2	Q8MRC9	521–643	X	X	X	X
UDP-α-N-acetylgalactosaminyltransferase 1	CAZyme	Yes	*Mus musculus*	AAB58477.1	O08912	426–551	X	X	X	X
*Ricinus communis* agglutinin	Lectin	Yes	*Ricinus communis*	AAA33869.1	P06750	309–436|439–563	X	X	X	X
Nigrin B	Lectin	Yes	*Sambucus nigra*	AAB39745.1	P33183	305–431|434–559	X	X	X	X
Abrin	Lectin	Yes	*Abrus precatorius*	AAF28309.1	Q9M6E9	292–419|422–546	X	X	X	X
Toxin Ha1	Lectin	Yes	*Clostridium botulinum* D phage CB16	BAA75077.1	P0DPR1	12–140|151–284	X	X	X	X
Hemolysin / cytolysin β-trefoil lectin VV20404	Lectin	Yes	*Vibrio vulnificus* CMCP6	AAO07360.2	P19247	338–465	X	X	X	X
Peptidase S1E	Other	Yes	*Stigmatella aurantiaca* DW4/3 − 1	ADO75988.1	E3FW82	390–516	X	X	X	X
1-phophatidylinositol phosphodiesterase	Other	Yes	*Lysinibacillus sphaericus*	CAL33524.1	A7WK54	351–485	X	X	X	X
β-1,4-N-acetylmuramidase	CAZyme	No	*Parascardovia denticolens* IPLA 20,019	EIT88793.1	I8UPP9	487–521|629–778|797–941	X	X	X	X
α-galactosidase	CAZyme	No	*Cordyceps militaris* CM01	ABA50434.1	G3JND5	439–546	X	X	X	X
α-1,2-mannosidase	CAZyme	No	*Rhodococcus* sp. AW25M09	CCQ17941.1	L8DMG7	56–185	X	X	X	X
β-agarase	CAZyme	No	*Catenovulum agarivorans* DS-2	EWH10233.1	W7QQS6	297–437	X	X	X	X
Endo-β-1,3-glucanase	CAZyme	No	*Amycolatopsis vancoresmycina* DSM 44,592	EOD68694.1	R1IE58	278–403	X	X	X	X
Ribosome inactivating protein	Lectin	No	*Iris hollandica*	AAL55094.1	Q8W2E7	337–463|466–591	X	X	X	X
Crystaline entomocidal toxin	Lectin	No	*Bacillus mycoides*	EJR29963.1	J8HQM8	662–808	X	X	X	X
HA-33 protein	Lectin	No	*Clostridium botulinum*	CAA74632.1	Q799J1	12–143|154–279	X	X	X	X
Lectin PVL	Lectin	No	*Streptomyces clavuligerus*	EDY49672.1	B5GTM6	6-154	X	X	X	X
Mosquitocidal toxin Mtx	Lectin	No	*Paenibacillus larvae*	ETK26578.1	W2E4N5	281–418|428–557	X	X	X	X
Peptidase M27	Other	No	*Cystobacter fuscus* DSM 2262	EPX55599.1	S9Q2U2	241–371|372–500	X	X	X	X
1-phophatidylinositol phosphodiesterase	Other	No	*Sphingobacterium spiritivorum* ATCC 33,861	EFK57648.1	D7VP01	314–453	X	X	X	X

For both groups of CBM13 proteins and putative ricin-B lectins, examples with CAZyme/lectin/other functionality are displayed, with taxonomical information, Genbank/UniProt ID, domain coordinates and ricin-B/CBM13 protein domain identifiers. The CBM13 and putative ricin-B lectin entries are described by the same protein domain identifiers

Previous observations demonstrate that CBM13 proteins and putative ricin-B lectins are indistinguishable based on the nomenclature used. The question arises whether or not it is still relevant to obstinately keep both groups of proteins apart, since both arguments in favour of and arguments against the merging of these protein groups are relevant. On one hand, both groups have been proven very similar on nomenclatural, structural and functional levels [4, 14]. Therefore, putative ricin-B lectins and CBM13s could be considered as distant members of one large ricin-B/CBM13 superfamily [14]. On the other hand, considering the carbohydrate-binding regions of ricin-B lectins as CBM13s, or the other way around, could lead to incorrect generalisations. Since the CAZy database includes both characterised proteins and proteins that show significant sequence homology towards characterised members, it may be relevant to separate the CAZy members from the non-CAZy members in public databases. Therefore, several ricin-B lectin domains are classified as CBM13, but certainly not every CBM13 module is considered a ricin-B lectin domain. Consequently, CBM13 modules may have been classified mistakenly as ricin-B lectin domain. It was observed that the CBM13 module is often not acknowledged in scientific reports. Examples include the ‘RICIN-like’ domain of a GH64 endo-β-1,3-glucanase from *Cellulosimicrobium funkei*, Ricin-B like lectins from *Saprolegnia parasitica* and *Hericium erinaceus*, whose carbohydrate-binding domains are in fact CBM13 modules but are not reported as such [72–74]. To keep the distinction between CAZy members and non-CAZy members visible in the public databases, several suggestions are described below.

Applied to the specific ricin-B/CBM13 case, confusions may be resolved by extension and revision of the CAZy cross-references, and the creation of a novel InterPro identifier dedicated to CBM13 modules (Table 3). The introduction of novel CBM identifiers may also be useful in other cases where there is overlap between lectin classification and CBM classification. Other examples include Hevein lectins versus CBM18, fucolectins versus CBM47/CBM51, malectins versus CBM57 and *Pleurotus* spp. lectins versus CBM67. New InterPro identifiers are released regularly [55], but only *n* = 23 identifiers are currently dedicated to CBM families.

Taking the aforementioned suggestions into account, CAZymes and other proteins with or without CBMs, classified in CAZy, would still be attributed with the current CAZy cross-reference and novel CBM identifiers, if applicable (Table 3, combinations 1, 2 and 3). CAZy also contains ricin-B lectins, which could be attributed with a CBM identifier, CAZy cross-reference and ricin-related identifiers (Table 3, combination 4). Proteins with a ricin-B lectin domain without CAZy membership, would only be attributed with ricin-related identifiers (Table 3, combination 5). Although, the introduction of a novel CBM InterPro domain identifier would provide clarity to some extent, it may cause problems for chimerolectins (Table 3, combinations 6 and 7).

**Table 3:** Potential solution for the ricin-B/CBM13 nomenclatural dichotomy

Combination Domain attribution	1	2	3	4	5	6	7
CBM13 module	X		X	X			X
CAZyme (i.e. CE, GH, GT, PL, AA) domain		X	X			X	X
Ricin-B lectin domain				X	X	X	X
**Corresponding semantics in UniProt/InterPro**							
CAZy cross-reference in UniProt	X	X	X	X		X	X
CBM13 identifier	X		X	X			X
Ricin-related domain identifiers				X	X	X	X

Depending on the databases certain entries belong to different protein domain identifiers and CAZy cross-references are applicable. Combinations 1–4 describe classical CAZymes and ricin-B lectins that are integrated in the CAZy database. Combination 5 describes ricin-B lectins not integrated in the CAZy database. Combinations 6–7 describe chimerolectins

Chimerolectins are fusion proteins that consist of a lectin domain and another non-lectin domain on the same polypeptide [6]. Several examples of chimerolectin sequences in various taxonomical lineages have been reported [33, 75–77]. Transcriptomics analyses in oysters have proven the expression of chimerolectins with catalytic activity, comprising of a ricin-B lectin domain or a Concanavalin-A lectin domain, in combination with a peptidase domain, amongst others [75]. Furthermore, chimerolectins with catalytic activity comprising of a ricin-related R-type lectin domain and GT27 domain have been functionally characterised [78].

Interestingly, in plants, multiple sequences of chimerolectins with catalytic GH domains have been identified, although only few have been characterised biochemically at present. Examples include combinations of GH1, GH5, GH17, GH19 and GH27 domains, in combination with Hevein and ricin-B domains [33]. In case a ricin-B chimerolectin with catalytic activity would be fully biochemically characterised, we suggest that it should be attributed with ricin-related domain identifiers and a cross-reference in UniProt towards the CAZy families involved (Table 3, combination 6). If the ricin-B chimerolectin would show high sequence homology towards CBM13 members, then also the CBM13 domain identifier would be appropriate (Table 3, combination 7). Chimerolectins containing domains with enzymatic activity complicate the classification of CBMs and lectins. In 1988, lectins were conceived as proteins different from antibodies and enzymes, which can bind carbohydrates reversibly [79]. This definition did not consider that lectins could also exist as chimerolectins with catalytic modules. Indeed, the definition of 1988 predated the discovery of chimeric lectins. After the discovery of several plant enzymes such as type 2-RIPs composed of a lectin domain (with two carbohydrate-binding sites) and a catalytic ricin-A domain [80, 81], the definition of what is considered as a lectin was too narrow and was updated. It was concluded that the definition of lectins should not exclude enzymatic activities completely. The currently accepted definition, as of 2018, states that proteins can be considered as lectins if the domain architectures involve at least one lectin domain which binds reversibly to carbohydrate structures without showing enzymatic activity, whether or not in combination with another protein domain [6, 82]. The current definition does not exclude catalytic chimerolectins as the nature of the other protein domain is not specified. However, one additional criterion for chimerolectins includes that the lectin domain has to act independently from the other protein domain(s) [32]. Interestingly, this definition of a catalytic chimerolectin shows many similarities to CAZymes being equipped with CBMs. It was already suggested that the occurrence of domains with enzymatic activity is not exclusively associated to CBMs [14]. However, CAZymes with CBMs should not be referred to as chimerolectins since the concept of ‘chimerolectins’ places the emphasis on the carbohydrate-binding activity rather than the catalytic activity. Furthermore, it was demonstrated before that the carbohydrate-recognition domain of CAZymes supports the activity of the catalytic domain [4, 83]. With respect to the ricin-B/CBM13 case, the nomenclature is complicated, in particular because the founding member of CBM13 was the ricin lectin. It is therefore debatable whether or not a novel chimeric ricin-B lectin with catalytic domain should be referred to as a catalytic ricin-B lectin or as a CAZyme with CBM module of family 13.

One particular example of a chimerolectin with CAZyme activity is a *Brassica juncea* chitinase 1, consisting of two chitin-binding domains belonging to the Hevein lectin family [84]. Hevein lectin domains are classified in the CAZy database as a member of CBM18 [85]. Chitinases are found in multiple CAZy families, including GH18 (inactive chitinases) and GH19 (active chitinases with chitinolytic activity) [86]. Since the Hevein domain is duplicated, investigation of hemagglutinating activity should be possible, although hemagglutination is no longer considered as a prerequisite to be recognised as a lectin [32, 87]. The *B. juncea* chitinase was expressed in transgenic potato and displayed clear hemagglutination and chitinase activity, rendering it as one of the first examples of fully characterised catalytic chimerolectins [84]. Recently, another functional chitinase with intact Hevein domain was identified from the tree *Simarouba glauca*. The Hevein domain shows a similar 3D structure (RMSD = 0.966 Å over 32 aligned residues; *calculated in this study*) and high sequence identity (57%) compared to the model lectin Hevein from *Hevea brasiliensis*. More importantly, the chitinase domain demonstrated significant enzymatic activity on insoluble chitin and against fungi [88]. However, the lectin properties of this particular protein were not reported.

### Characteristics of the ricin-B/CBM13 SSN

The reference set of compiled CBM13 modules comprised *n* = 7963 individual protein sequences. BLASTp of the sequence space against the compiled CBM13 database yielded *n* = 51,715 hits. After removing duplicate modules, ranking of modules by prediction quality and fivefold reduction, a total of *n* = 8976 modules was retained. The SSN covers 9.9% of the initial ricin-B/CBM13 sequence space and comprises of 12.9% CBM13 (*n* = 1158) and 87.1% putative ricin-B lectin (*n* = 7818) entries. The corresponding network file and nodes list is given in Supplementary File S5 and Supplementary File S6 respectively.

SSNs show clustering based on sequence similarities, thereby mimicking clade formation as seen in traditional phylogenetic trees [40]. The clusters in each SSN are ordered by decreasing size (i.e. number of edges and nodes). Depending on the threshold E-value, the size of the SSN varied greatly, as well as the number and shape of clusters within each SSN (Table 4). The SSN at threshold E = 10^− 100^ is considerably more concise and contained many small and isolated clusters, while the SSN at less stringent threshold E-values, for instance at E = 10^− 40^, E = 10^− 30^ and E = 10^− 20^ were characterised with larger and more complex looking clusters with less isolated modules. In the SSN at threshold E = 10^− 100^ no main cluster was identified as the SSN only contained small subclusters (Supplementary File S7).

In each SSN, the distance between the modules represents the pairwise alignment score. A property of SSNs is that intra-cluster sequence similarity is higher than inter-cluster sequence similarity, indicating that nodes belonging to the same cluster are phylogenetically related, while neighbouring clusters are not necessarily related. Because of practical reasons concerning computing time and power, a threshold E-value of 10^− 30^ was used to perform the analyses in Cytoscape, thereby retaining approximately 99% of the initially considered modules (Table 4) (Supplementary File S8).

**Table 4:** Size of the SSN at varying E-values

E-value threshold	Number of edges	Number of nodes	Retained modules in SSN (%)
10^− 100^	5842	2472	27.5
10^− 80^	46,885	7519	83.8
10^− 60^	147,149	8355	93.1
10^− 40^	651,519	8789	97.9
10^− 30^	1,491,484	8892	99.1
10^− 20^	3,631,702	8957	99.8
10^− 15^	5,653,113	8970	99.9
10^− 10^	9,558,588	8976	100.0
10^− 5^	15,092,062	8976	100.0

**Fig. 7:**
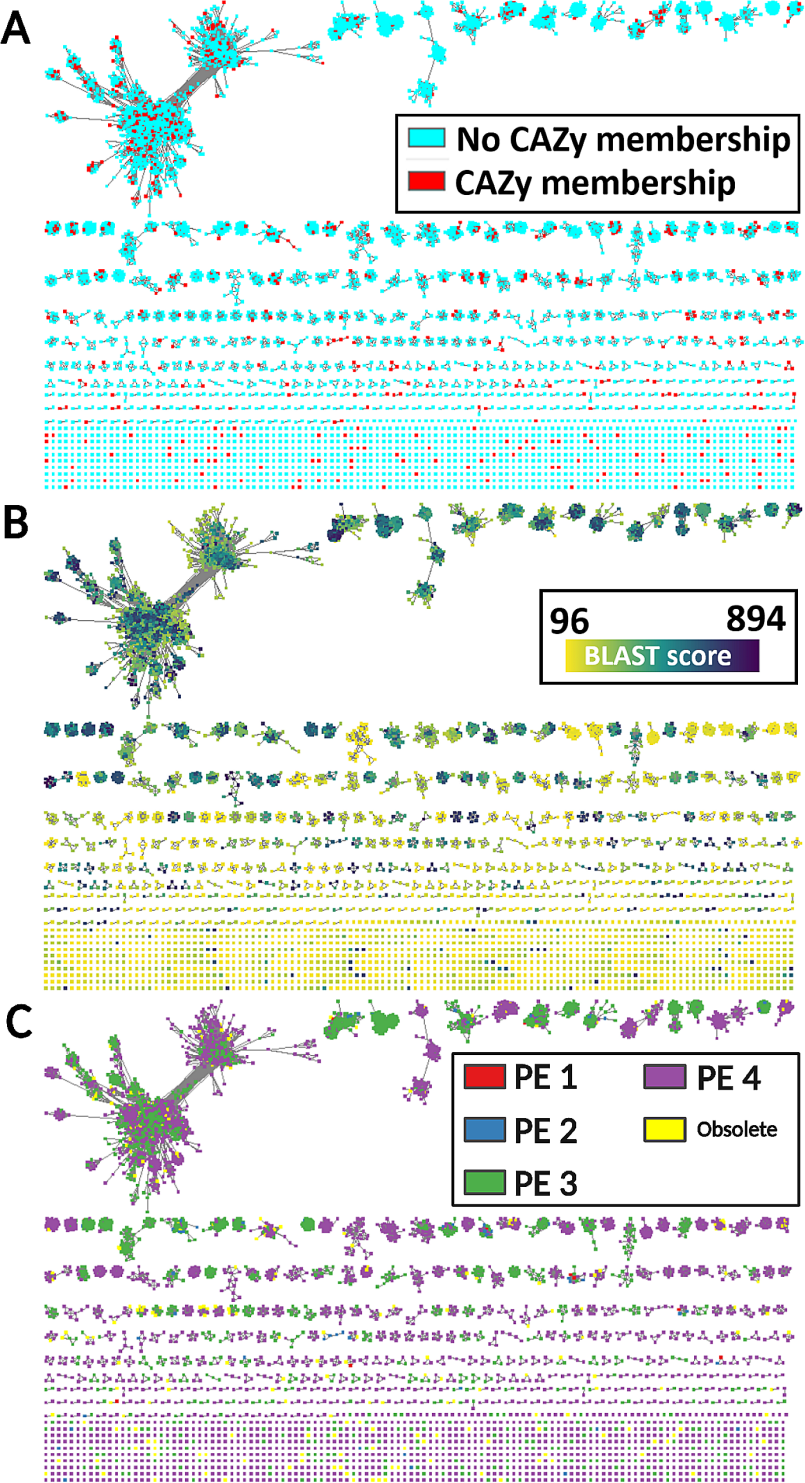
SSN at threshold E = 10^− 30^ overlaid with metadata. **A**: CAZy membership is uniformly distributed across the SSN. **B**: module prediction BLAST scores are proportional to the number of modules per cluster because of reciprocal comparison. **C**: protein existence (PE) levels are not distributed randomly over the SSN and are mostly predicted or inferred from homology. Some modules were already obsolete at the moment of data analysis

Different biological metadata of the CBM13 modules are projected onto the SSN (Table 5). The first investigated characteristic is the spatial distribution of CBM13 modules and CBM13-predicted ricin-B lectin modules across the SSN. Modules from both groups are distributed evenly across every cluster from the SSN, rather than being confined to separate clusters (Fig. 7A). This implies that the CAZy database contains a wide diversity of modules, showing great high variability at amino acid sequence level and is certainly not restrained to one particular type of proteins. Moreover, it also implies that several CBM13 modules show high sequence similarity towards ricin-B lectin modules. Noteworthy, there are only very few clusters without a CBM13 module. Furthermore, there are also several isolated modules, from both SSN subdivisions, which are not showing sequence similarity to any other module.

The SSN at threshold E = 10^− 30^ contains modules with BLAST scores ranging between 96 and 894. The higher the BLAST score, the more trustworthy the prediction of the CBM13 module in the putative ricin-B lectin entries. Interestingly, mostly modules with low BLAST scores are observed in the clusters with lower numbers of modules. Because of low reciprocal similarity, these modules are usually presented as non-clustered and isolated entities. The larger clusters usually contain modules with intermediate or higher BLAST scores (Fig. 7B). As expected, CBM13-predicted ricin-B lectin modules have significantly lower BLAST scores compared to CBM13 modules (Welch’s t = -19.051; ${v}$ = 1557 df; one-sided *p* < 0.001) (Table 5). Most modules (95%) are derived from proteins with PE level 3 (existence inferred from homology) or PE level 4 (predicted). The CBM13 subspace contains more sequences with PE levels 1 (evidence at protein level) or PE level 2 (evidence at transcript level), compared to the putative ricin-B lectin subspace (${X^2}$ = 209.975; ${v}$ = 3 df; one-sided *p* < 0.001). Visually, it is clear that PE level distribution in the SSN is not random as most clusters contain modules derived from proteins of the same PE level (Fig. 7C). Many modules of PE level 4 (predicted) arise from sequences without an experimentally characterised close relative. Although these modules should be considered taxonomically and biochemically relevant, selection of sequences from UniProt for further investigations, especially sequences with PE level 4, should be carried out carefully with attention for sequence quality.

**Fig. 8:**
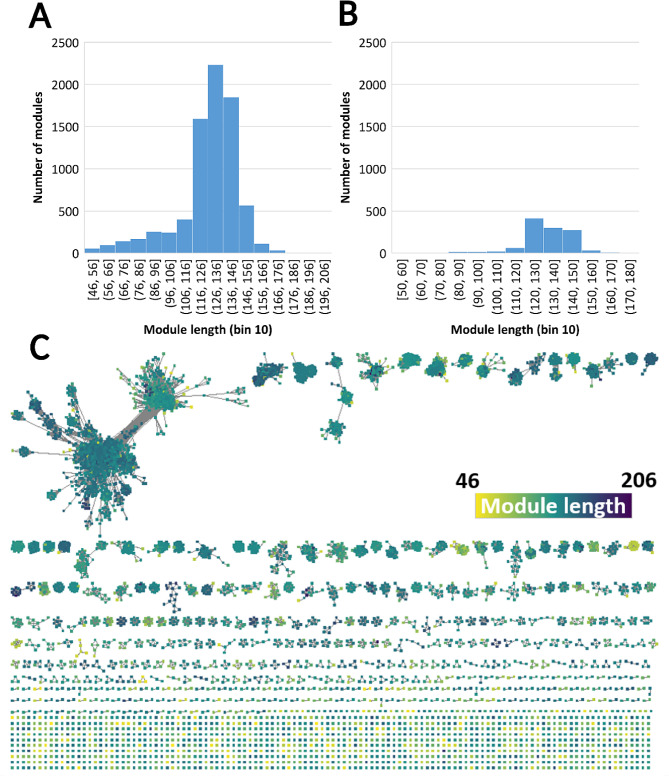
Distribution of module length across the SSN at threshold E = 10^− 30^. **A and B**: histograms, bin size 10 of CBM13-predicted ricin-B lectin modules (**A**) and CBM13 (**B**) modules length distribution. **C**: modules coloured by module length on a discreet scale depicting shorter (yellow) and longer (blue) modules

Across the SSN, module length of CBM13 modules and CBM13-predicted ricin-B lectin modules ranged between 46 and 206 amino acids, although most of the modules were more centered around the average length of 128 ± 19 residues (Fig. 8A and B). Visually, there is no apparent clustering based on module length (Fig. 8C), although most shorter modules appear where modules with low BLAST scores were observed earlier. Finally, many of the short (< 60 residues) modules appear as isolated clusters and mostly have PE level 4 scores (predicted). These isolated clusters cannot not have a trefoil structure like bona fide CBM13s and are likely to be artefacts and/or false positives. Significant correlations were calculated between the module length and the number of QXW motifs (Pearson’s *r* = 0.193; two-sided *p* < 0.001), and the module length and the BLAST E-value (Pearson’s *r* = -0.250; two-sided *p* < 0.001).

The modules contained on average 2.3 ± 1.3 QXW motifs, although the majority (58%) contained only 1 QXW motif. It should be pointed out that other aromatic amino acid residues different from tryptophan can also be involved in protein-carbohydrate interactions [89], although the preference for tryptophan is ninefold higher [90]. Therefore, if tyrosine (QX*Y*) and phenylalanine (QX*F*) residues are also considered (i.e. QX[F; W;Y]) the number of possible interaction sites with carbohydrates would increase to an average of 3.1 ± 1.5.

On average, CBM13-predicted ricin-B lectin modules are shorter compared to CBM13 modules (Welch’s t = -10.171; ${v}$ = 1897 df; one-sided *p* < 0.001), and contain less QXW motifs (Welch’s t = -8.788; ${v}$ = 1494 df; one-sided *p* < 0.001) and QX[F; W;Y] motifs (Welch’s t = -8.708; ${v}$ = 1487 df; one-sided *p* < 0.001) (Table 5). However, it should be mentioned that the number of QX[F; W;Y] motifs itself is not a good proxy for the carbohydrate-binding capabilities of a protein, since also structural aspects like positioning of the binding site towards the protein’s surface should be considered. Obviously, QX[F; W;Y] motifs oriented towards the core of the protein cannot participate in protein-carbohydrate interactions [91]. Furthermore, it should be stressed that the BLAST algorithm has an intrinsic tendency to predict shorter modules, which also influences the number of carbohydrate-binding sites that can be expected in predicted modules [92]. Indeed, small scale module length comparison of *n* = 20 random CBM13-predicted ricin-B lectin modules to the InterPro-predefined module length, revealed that most predicted modules are indeed shorter than the modules predicted in InterPro. Moreover, at least one CBM13-predicted ricin-B lectin module (UniProt ID: A3Y6T7) was truncated after performing BLAST. The truncated end of the module contained an additional QX[F; W;Y] (Supplementary File S9). Therefore, apparent differences in module length and number of QX[F; W;Y] motifs cannot be considered unilaterally as distinctive characteristics of predicted ricin-B lectin versus CBM13 modules.

**Table 5:** Comparison of BLAST scores, module length and QXW/QX[F; W;Y] motifs of CBM13 and CBM13-predicted ricin-B lectin modules

		SSN at threshold E = 10^− 30^	CBM13-predicted ricin-B lectin modules	CBM13 modules
BLAST score	Average ± sd	429.1 ± 206.0	414.0 ± 204.0	530.5 ± 191.7
	Median	448.0	430.0	580.0
	Range	96–894	96–894	106–894
Module length	Average ± sd	128 ± 19	127 ± 20	132 ± 14
	Median	130	130	132
	Range	46–206	46–206	50–174
QXW motifs	Average ± sd	2.3 ± 1.3	2.3 ± 1.3	2.6 ± 1.3
	Median	2.0	2.0	3.0
	Range	0–6	0–6	0–6
QX[F; W;Y] motifs	Average ± sd	3.1 ± 1.5	3.0 ± 1.5	3.5 ± 1.5
	Median	3.0	3.0	3.0
	Range	0–8	0–7	0–8

In the SSN at threshold E = 10^− 30^, CBM13-predicted ricin-B lectin modules are usually shorter with fewer QXW and QX[F; W;Y] motifs compared to CBM13 modules. Abbreviation: sd (standard deviation)

### Taxonomical composition of the ricin-B/CBM13 SSN

**Fig. 9:**
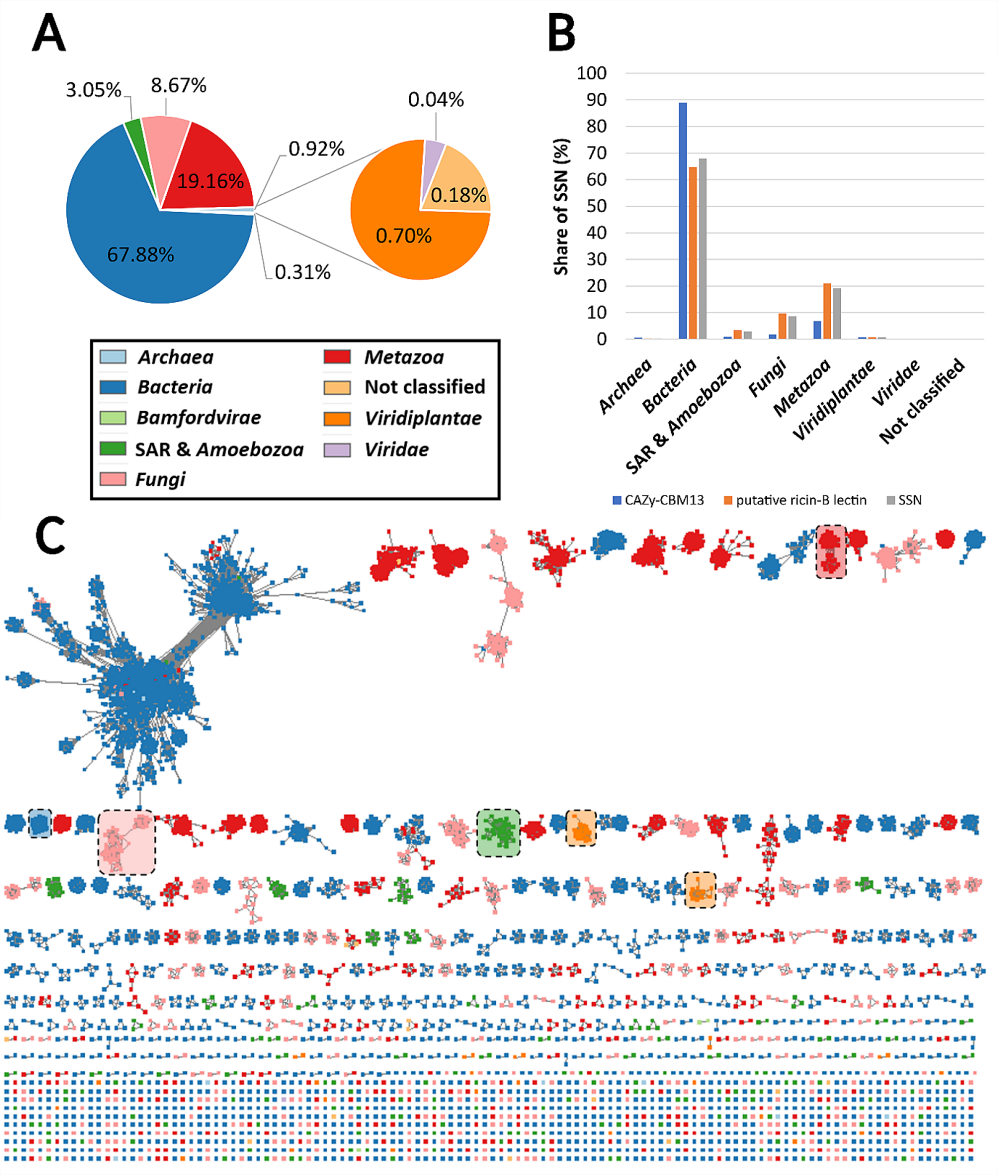
Kingdom-wide distribution of CBM13 and putative ricin-B lectin modules. **A** and **B**: The ricin-B/CBM13 entries are mainly found in prokaryotes and *Metazoa*, and to a lesser extent in *Fungi* and *Viridiplantae*. **C**: Each colour represents a different taxonomical kingdom: blue (*Bacteria*), red (*Metazoa*), pink (*Fungi*), orange (*Viridiplantae*) and green (SAR and *Amoebozoa*). The highlighted groups are example clusters of organisms belonging to one particular taxonomic group

The SSN at threshold E = 10^− 30^ covers modules originating from all kingdoms of life. Most modules originated from *Bacteria* (67.9%), followed by *Metazoa* (19.2%), *Fungi* (8.7%), SAR (*Stramenopiles-Alveolata-Rhizaria*) and *Amoebozoa* (3.1%), *Viridiplantae* (0.7%) and *Archaea* (0.3%) (Fig. 9A). Both SSN subdivisions were mainly represented by bacterial modules. The CBM13 subdivision shows slight overrepresentation of bacterial modules, as almost 90% of the modules are of bacterial origin, compared to approximately 65% bacterial modules in the putative ricin-B lectin subdivision. In contrast, the putative ricin-B lectin subdivision shows slight overrepresentation of modules from *Fungi*, *Metazoa*, SAR and *Amoebozoa* (Fig. 9B). Looking at the topological level, the different modules principally associate in clusters based on taxonomic origin. The largest cluster contains mainly modules from *Bacteria* with a few outliers from the *Metazoa* and *Viridiplantae* (Fig. 9C).

### Functional characteristics of the ricin-B/CBM13 SSN

**Fig. 10:**
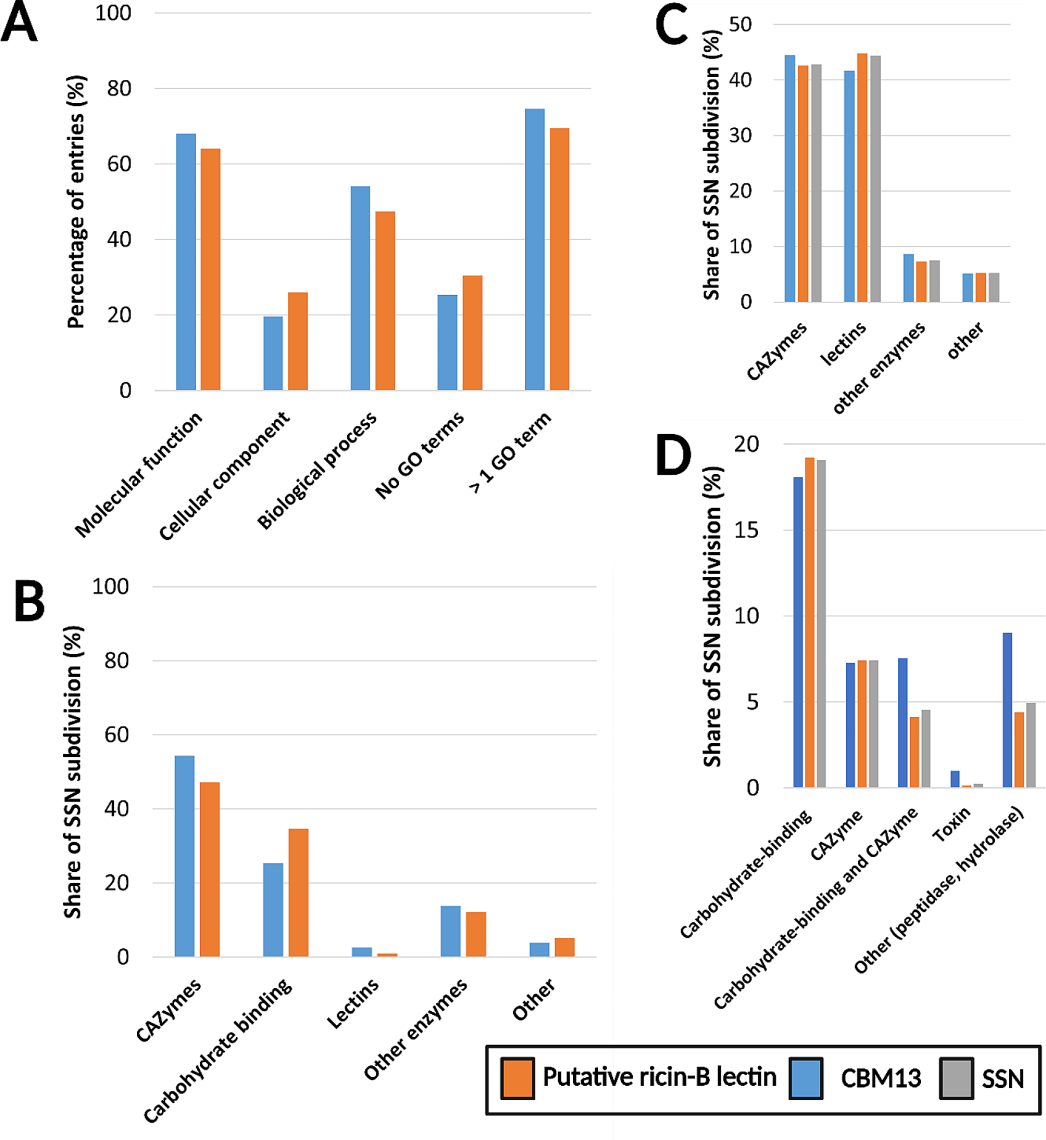
Analysis of GO terms and protein names. **A**: Distribution of GO levels among the CBM13 and putative ricin-B lectin entries shows that at least 69% of all entries are equipped with one GO term. **B**: Distribution of different types of molecular functions reveals that the majority is attributed with GO terms related to CAZyme activity and carbohydrate-binding activity. **C**: Distribution of protein name categories ‘CAZymes’, ‘lectin related’, ‘other enzymes’ and ‘other’ shows that the majority of entries is related to CAZyme or lectin related protein. **D**: Distribution of ricin-related entries over different activities represented by GO terms in the CBM13 and putative ricin-B lectin SSN subdivisions

GO terms belong to three possible categories: ‘molecular function’, ‘cellular component’ or ‘biological process’. The majority of the CBM13 (74.6%) and putative ricin-B lectin (69.5%) subdivision was equipped with at least one GO term, although not every GO category was represented (Fig. 10A). The GO category ‘molecular function’ describes the activity a protein fulfils based on traceable and proven experimental data [93, 94] and occurred most frequently in both CBM13 (68%) and putative ricin-B lectin (64.1%) subdivisions. The 20 most frequent GO terms in both subdivisions are shown in Supplementary File S10. Analysis of GO term distribution reveals that the majority of CBM13 and putative ricin-B lectin entries are associated with CAZyme activity (54.4% and 47.1% respectively), followed by carbohydrate binding activity (25.5% and 34.6% respectively) (Fig. 10B). Reoccurring enzymatic activities in both SSN subdivisions are related to GHs and GTs, including α-L-arabinofuranosidase activity, polypeptide: N-acetylgalactosaminyl-transferase activity, endo-1,4-β-xylanase activity and raffinose α-D-galactosidase activity. In addition, other enzymatic activities were found, mostly metalloendopeptidases and serine-type endopeptidases. References to lectins occurred less frequently, for instance rRNA: N-glycosidase activity and toxin activity, both referring to typical characteristics of type-2 RIPs [8].

Analysis and categorisation of protein names reveals a remarkable entry distribution, different from the GO terms analysis. After assigning entries to one of the four protein name-based categories (i.e. ‘CAZymes’, ‘lectin related’, ‘other enzymes’ and ‘other’), revealed that the distribution across SSN subdivisions was very similar, without significant differences (${X^2}$ = 4.767; ${v}$ = 3 df; two-sided p = 0.190), as shown in Fig. 10C. Meaning that, based on protein names, comparable proportions of CAZymes, lectins and enzymes with other activities are expected in both subdivisions. Recurring CAZyme names included β-1,4-xylanases, β-1,3/1,4-glucanases, α-D-galactosidases, α-L-arabinofuranosidases, β-xylosidases and polypeptide: N-acetylgalactosaminyl-transferases. The lectin-related entries were predominantly referring to ricin-B lectins. In the category ‘other enzymes’, various enzymatic activities were present, such as peptidases, protein kinases, (de)hydrogenases and lipases. The last category ‘other’ contained entries that could not be classified into one of the first three categories, including entries with names like ‘secreted protein’, ‘uncharacterised protein’, ‘transmembrane protein’, ‘secreted protein’ or other (Supplementary File S11).

Grouping of SSN entries by protein name, revealed that the majority (*n* = 3412) of the CBM13 and putative ricin-B lectin entries was named after the ricin-B domain, probably due to automatic annotation. Surprisingly, the proportion of ricin-related protein names was almost equal amongst CBM13 (34.4%) and putative ricin-B lectin (38.7%) entries. Surprisingly, only 43% of CBM13 and 35.3% of putative ricin-B lectin entries with a specific ricin-related name is attributed with a GO term related to molecular functions, including CAZyme-related names, peptidases or other hydrolases (EC 3.-.-.-) (Fig. 10D).

Furthermore, the GO terms from CBM13 and putative ricin-B lectin entries with ricin-related names were not distributed evenly (${X^2}$ = 26.239; ${v}$ = 4 df; two-sided *p* < 0.001) over the aforementioned categories (i.e. ‘CAZymes’, ‘lectin related’, ‘other enzymes’ and ‘other’). Hence, specific GO terms occur more frequent in one SSN subdivisions compared to others. This significant difference could not be attributed to the GO terms related to ‘carbohydrate-binding’ or ‘CAZyme’ activity separately (${X^2}$ = 0.030; ${v}$ = 1 df; two-sided p = 0.863), meaning that both SSN subdivisions contain similar proportions of entries with either ‘carbohydrate-binding’ (18.1% and 19.2% respectively) or ‘CAZyme’ (7.3% and 7.4% respectively) activity related GO terms. In contrast, the CBM13 SSN subdivision contains more entries attributed with GO terms related to both ‘carbohydrate-binding’ and ‘CAZyme’ activity (${X^2}$ = 8.199; ${v}$ = 2 df; one-sided p = 0.0085).

Entry categorisation based on protein names is not fool-proof, may be ambiguous and prone to biases since a protein name is no guarantee for its biological function. Very often, proteins are named after the module that shows the highest degree of homology to, although this is strongly discouraged by the International Protein Nomenclature Guidelines [95]. Therefore, also GO terms should be considered since these are based on experimental data [93].

The differences between the GO term analysis and protein name categorisation indicate a discrepancy between the assigned protein name and the functionality based on attributed GO terms. However, for the majority of the entries in the SSN at threshold E = 10^− 30^, GO terms and protein names are in agreement. For instance, the β-xylanase (UniProt ID: Q9 × 584) from *Streptomyces avermitilis* is foreseen with the GO term of endo-1,4-β-xylanase activity.

**Fig. 11:**
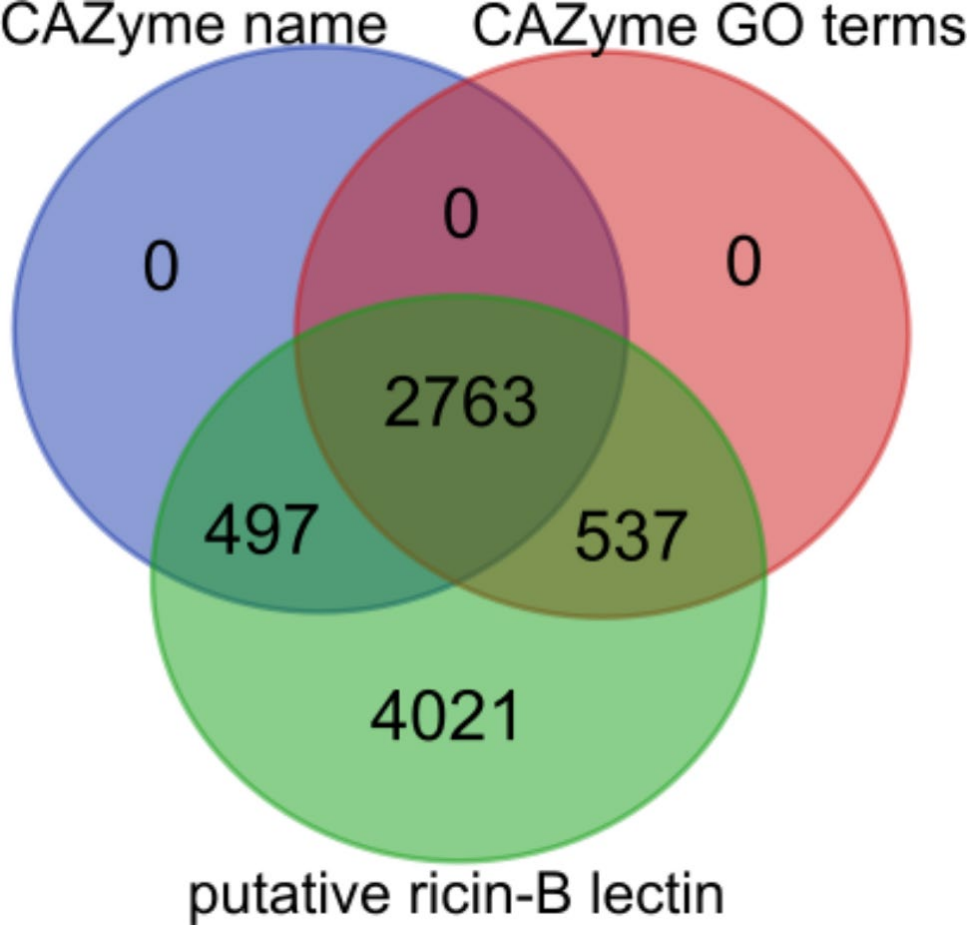
Venn diagram comparing putative ricin-B lectin entries with possible CAZyme functionality. Approximately half of the putative ricin-B lectin entries have a protein name and/or GO terms indicating CAZyme functionality

However, it is apparent that several entries show a difference between the assigned protein name and GO terms. Remarkably, many of the putative ricin-B lectin entries with ricin-related protein names, are attributed with GO terms unrelated to lectins. For instance, the protein name of ‘Ricin B lectin domain-containing protein’ (UniProt ID: A7RJ47) from *Nematostella vectensis* suggests the protein to be a ricin-B lectin. However, since the GO term ‘polypeptide: N-acetylgalactosaminyltransferase activity’ was attributed, this protein is most likely a GT equipped with CBM13 module, and not a ricin-B lectin. Almost half (48.6%; *n* = 3797) of all putative ricin-B lectin entries in the SSN at threshold E = 10^− 30^ have protein names or GO terms that suggest CAZyme functionality (Table 6). Close to one third (35.3%; *n* = 2763) of the putative ricin-B lectin entries have both a CAZyme-related protein name and GO terms (Fig. 11). This group encompasses eligible candidates to further investigate possible CBM13 membership (Supplementary File S11).

**Table 6:** Many putative ricin-B lectin entries have protein names and GO terms suggesting CAZyme functionality

CAZy category	CAZyme functionality	Count GO-term based	Count protein name based
AA	Galactose oxidase	1	5
AA	Gluco-oligosaccharide oxidase	0	2
CE	Feruloyl esterase	64	3
CE	Pectate/pectin esterase	14	13
GH	α-amylase	4	6
GH GH + GH GH + CE	α-L-arabinofuranosidase α-L-arabinofuranosidase + (endo-)β-1,4-xylanase α-L-arabinofuranosidase + feruloyl esterase	236 26 6	237 0 0
GH	(endo-)α-L-1,5-arabinosidase	6	38
GH	α-L-fucosidase	58	56
GH	α-D-galactosidase (melibiase)	170	210
GH	α-glucosidase	0	5
GH	α-D(-1,2/1,4)-mannosidase	0	20
GH	α-trehalase	9	0
GH	(endo-)α-1,4-polygalactosaminidase	0	2
GH	α-L-rhamnosidase	0	1
GH	β-agarase	3	5
GH	(endo-)β(-1,4)-galactosidase	35	36
GH	(endo-)β(-1,4)-galactanase	0	12
GH	(endo-)β(-1,3/1,4)-D-glucanase	0	126
GH	(endo-)β(-1,3/1,4/1,6-)glucosidase	16	32
GH	β-fructosidase	0	7
GH	(endo-)β-1,4-mannosidase	20	0
GH	β-1,4-N-acetylmuramidase (lysozyme)	21	8
GH GH + GH	β-N-acetylhexosaminidase β-N-acetylhexosaminidase + β-N acetylgalactosaminidase	2 17	12 0
GH	(exo-)β-1,4-D-glucosaminidase	1	2
GH GH + CE	(endo-)β-1,4-xylanase (endo-)β-1,4-xylanase + feruloyl esterase	108 3	186 0
GH	β-1,4-xylosidase	1	103
GH	Arabinanase	0	5
GH	Cellulase	21	38
GH	Chitinase	22	58
GH	Chitosanase	14	4
GH	Cutinase	2	0
GH	Dextranase	0	2
GH	Galactosidase	39	0
GH	Mannanase	0	4
GH	Galactosylceramidase	45	43
GH	Glucosylceramidase	130	81
GH	Lacto-N-biosidase	0	1
GH	Levanase	0	8
GH	Licheninase	7	2
GH	κ-carrageenase	3	3
PL	Alginate lyase	0	4
PL	Pectate/pectin/polysaccharide lyase	58	67
PL	Rhamnogalacturonan lyase	0	5
GT	Polypeptide: N-acetylgalactosaminyltransferase	581	1216
GH	Non-specified GH	965	442
GT	Non-specified GT	589	26
PL	Non-specified PL	4	0
CBM	Carbohydrate binding module	0	124
Total		3301	3260

Most likely, these ricin-related protein names were assigned automatically based on the presence of a QXW motif, which was first reported for ricin-B lectins [13]. The given examples (Table 6) show that protein names alone are a naïve proxy for biological function and introduce a significant bias towards ricin-B lectins. Protein names are often arbitrarily or automatically assigned and may uphold a discrepancy with their proposed biological function. Furthermore, it should be noted that CBM13 membership should not depend solely on protein names and GO terms indicating a possible molecular function. However, a criterium for CAZy membership includes the sequence homology to at least one characterised CBM13 member. Finally, biological evidence for proposed CAZyme functionality remains crucial for CAZy membership [1].

### CBM13 and ricin-B lectin modules are structurally similar

In this section, phylogenetic and structural similarities between CBM13 and CBM13-predicted ricin-B lectin modules were analysed. We manually selected individual example clusters from different taxonomical origins (Fig. 9C) with different enzymatic/protein activities (Table 7) from the SSN at threshold E = 10^− 30^. One exception was made for the *Viridiplantae* kingdom, where two clusters were joined to obtain a larger sample set (Supplementary File S12).

**Table 7:** Composition of the different example clusters in SSN at E = 10− 30

Taxonomical origin	Proposed function	Cluster size	Number of CBM13 modules	Number of putative ricin-B lectin modules
*Metazoa*	Polypeptide: N-acetylgalactosaminyl transferase	80	2	78
*Bacteria*	α-L-fucosidase	55	7	48
*Fungi*	α-D-galactosidase	50	2	48
*Viridiplantae*	Ribosome inactivating protein	35	9	26
SAR	Ricin-B lectin domain containing protein	33	2	31

**Fig. 12:**
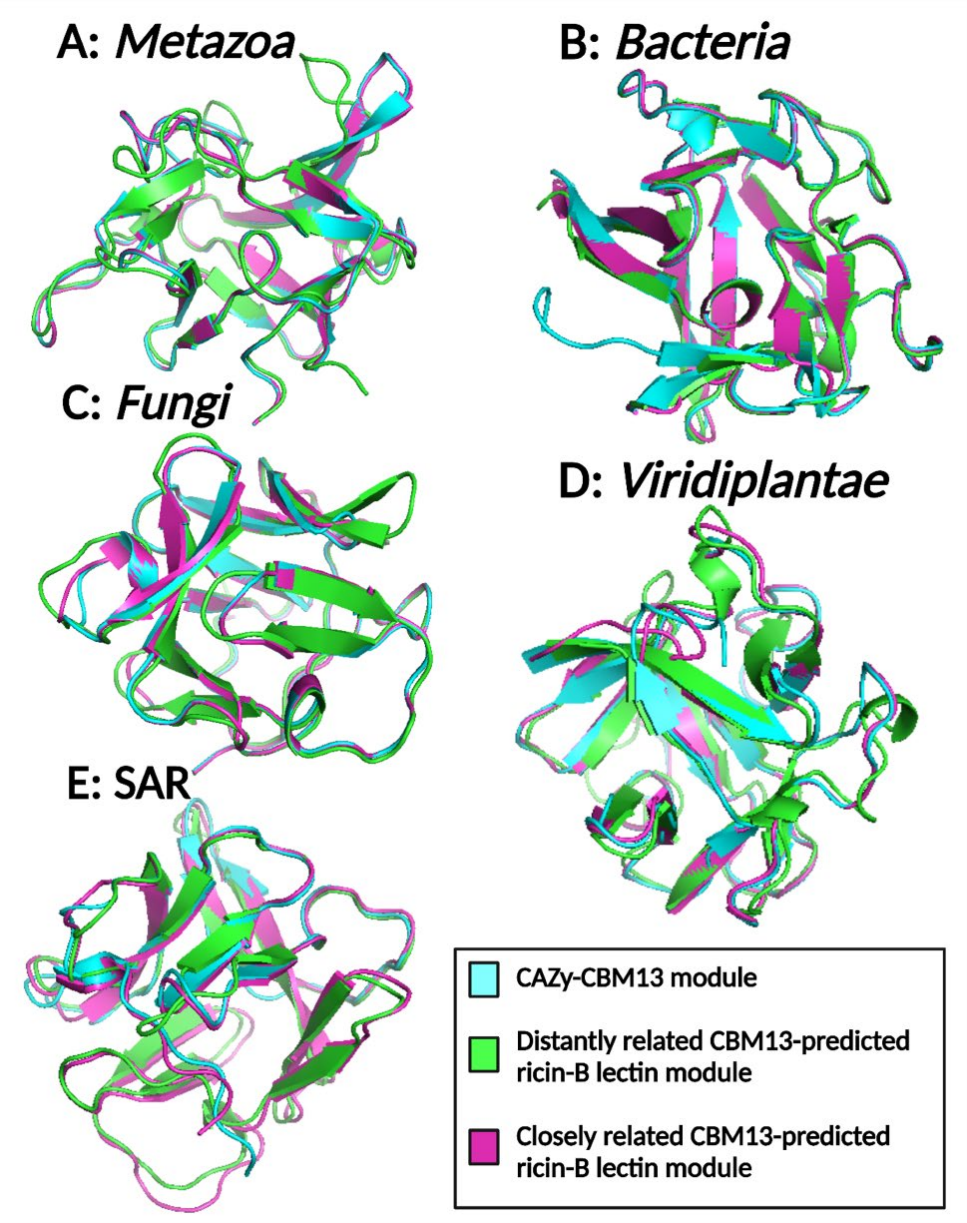
Structural alignment of AlphaFold models of CBM13 and CBM13-predicted ricin-B lectin modules from various taxonomical origins. **A**: Metazoan CBM13-predicted modules occurring in polypeptide: N-acetylgalactosaminyltransferases from *Phyllostomus discolor* and *Perca fluviatilis* versus CBM13 *Rattus norvegicus*. **B**: Bacterial CBM13-predicted modules occurring in α-L-fucosidases from *Streptomyces afghaniensis* and *Micromonospora humi* versus CBM13 *Streptomyces bingchenggensis*. **C**: Fungal CBM13-predicted modules occurring in α-D-galactosidases from *Aspergillus transmontanensis* and *Glarea lozoyensis* versus CBM13 *Aspergillus flavus*. **D**: Plant CBM13-predicted modules occurring in RIPs from *Tanacetum cinerariifolium* and *Cinnamomum micranthum* versus CBM13 *Sambucus nigra*. **E**: SAR CBM13-predicted modules occurring in ricin-B containing proteins from *Saprolegnia diclina* and *Thraustotheca clavata* versus CBM13 *Achlya hypogyna*

The *Metazoa* cluster comprises *n* = 80 (predicted) CBM13 modules present on the same polypeptides of polypeptide: N-acetylgalactosaminyl transferases. In the phylogenetic tree of *Metazoa*, two major clades are present (Supplementary File S12A). This is consistent with the shape of the SSN, since the selected cluster is composed of two subclusters that would be completely separated in an alternative SSN with more stringent E-value threshold. However, both clades and subclusters contain modules from the classes of birds (*Aves*), ray-finned fishes (*Actinopterygii*), cartilaginous fishes (*Chondrichthyes*) and mammals (*Mammalia*). None of the classes are confined to one clade or subcluster. A similar observation can be made for the *Fungi* (Supplementary File S12C) and *Viridiplantae* (Supplementary File S12D) clusters. The *Viridiplantae* cluster comprises *n* = 35 modules, although these originate from two joined subclusters. Within the *Viridiplantae* cluster, members from very diverse phylogenetic divisions, ranging from monocots (*f.i. Polygonatum multiflorum*), eudicots (*f.i. Camellia sinensis*) to Magnoliids (*f.i. Cinnamomum micranthum*) are present. Similarly, the *Fungi* cluster shows two large clades which mostly coincide with the nearly separated clusters as observed in the SSN topology (Fig. 9C). One fungal subcluster is mainly confined to the class of *Sordariomycetes*, while the other fungal subcluster contains other classes from the *Pezizomycotina* subdivision. The SAR cluster contains only *n* = 33 modules from the *Stramenopiles* clade (Supplementary File S12E). The *Bacteria* cluster contains *n* = 55 modules from the *Actinomycetia* class, mainly *Streptomyces* spp. and occasionally *Amycolatopsis* spp. and *Nonomuraea* spp. (Supplementary File S12B). Across all selected clusters, a high degree of sequence conservation was observed (Supplementary File S13). As expected, the typical QXW motifs and cysteine residues are most conserved [13]. However, several example clusters (*i.e. Metazoa* and *Bacteria*) show higher degrees sequence conservation than others. The chosen clusters are very diverse in taxonomic and phylogenetic background and contain module sequences from multiple taxonomic families. This highlights that CBM13 modules and ricin-B lectin domains are highly conserved per cluster, regardless of their phylogenetic distance. From each example cluster, one CBM13 and two CBM13-predicted ricin-B lectin modules were selected (Table 7). The CBM13-predicted ricin-B lectin modules were either closely or distantly related to the CBM13 module, based on the phylogenetic trees (Supplementary File S14) and percentual identity scores. Closely related modules showed higher identity scores, as expected (Table 8). In the SAR cluster, the difference in identity scores between the closely and distantly related module was not outspoken since the modules from this cluster all belonged to the *Stramenopiles* clade. In the other clusters, the phylogenetic distances were more important. Furthermore, CBM13 modules and CBM13-predicted ricin-B lectin modules are structurally very similar (Fig. 12). RMSD values as low as 0.13 Å were obtained, indicating a close structural alignment. Despite comparing remotely and distantly related modules, RMSD values below the threshold value of 2.00 Å were obtained (Table 8). The CBM13-predicted ricin-B lectin modules arose from non-CAZy sequences that were considered too different to be included in the CAZy database. However, the presented structural comparisons clearly demonstrate their structural similarity (Fig. 12). Since similar protein structures infer similar functions, it is very likely that the CBM13-predicted ricin-B lectin modules will display similar carbohydrate-binding properties as the CBM13 modules. The predicted catalytic activity of the adjacent CAZyme domains need to be proven experimentally in order to classify the putative ricin-B lectins in GH/GT/CE/AA families. The conclusions derived from the presented structural alignments are not anecdotical but can be expanded to all SSN clusters with CBM13-predicted ricin-B lectin modules and CBM13 modules.

Based on previous observations, it can be concluded that many of the putative ricin-B lectin modules are structurally similar to CBM13 modules. We demonstrated that even in distantly related modules, structural resemblances are still very high. The reason why these putative ricin-B lectin modules are not incorporated in CAZy is because CAZy enforces sequence homology-based categorisation rather than structure-based categorisation. It is, however, very likely that the CBM13-predcited ricin-B lectin modules will display a similar carbohydrate-binding activity since this activity is determined primarily by the structure of the protein domain [96]. Therefore, we conclude that the CBM13 family may be larger than initially envisaged.

**Table 8:** Phylogenetic and structural comparison of CBM13 and CBM13-predicted ricin-B lectin modules from different example clusters of SSN at threshold E = 1e-30

Function	Taxonomy			Entry identifiers		Prediction of CBM13 module from putative ricin-B lectins					Phylogenetic relatedness compared to CBM13	Structural comparison to CBM13		
	Kingdom	Division	Specimen	Genbank	UniProt	CAZy member	CBM13 module coordinates	BLAST score	QXW motifs	QX[F; W;Y] motifs		Identity (%)	RMSD (Å)	Aligned residues
polypeptide: N-acetyl galactosaminyl- transferase	*Metazoa*	*Mammalia*	*Rattus norvegicus*	BAD93348.1	Q58A68	Yes	442–578	764	1	2	*a*			
		*Mammalia*	*Phyllostomus discolor*	KAF6118728.1	A0A6J2NAZ3	No	442–577	717	1	2	Close	86.8	0.131	136
		*Actinopterygii*	*Perca fluviatilis*	KAF1381539.1	A0A6A5ET19	No	437–576	573	1	3	Far	34.6	1.713	136
α-L-fucosidase	*Bacteria*	*Actinomycetia*	*Streptomyces bingchenggensis*	ADI13044.1	D7CDK2	Yes	605–743	720	1	1	*a*			
		*Actinomycetia*	*Streptomyces afghaniensis*	EPJ40152.1	S4N0V9	No	596–743	671	1	1	Close	86.4	0.252	128
		*Actinomycetia*	*Micromonospora humi*	SCG33948.1	A0A1C5GJH6	No	528–658	508	1	1	Far	70.2	0.471	128
α-D-galactosidase	*Fungi*	*Eurotiomycetes*	*Aspergillus flavus*	QMW26614.1	A0A7G5ITC2	Yes	400–516	350	2	3	*a*			
		*Eurotiomycetes*	*Aspergillus transmontanensis*	KAE8312064.1	A0A5N6VTV9	No	233–349	349	2	3	Close	97.4	0.279	122
		*Leotiomycetes*	*Glarea lozoyensis*	EHL03104.1	H0EEC1	No	367–491	301	2	2	Far	41.9	1.222	122
Ribosome inactivating protein	*Viridiplantae*	*Asterids*	*Sambucus nigra*	AAC15886.1	O04367	Yes	299–436	752	3	3	*a*			
		*Asterids*	*Tanacetum cinerariifolium*	GEU49319.1	A0A6L2KK95	No	157–286	337	2	2	Close	44.2	0.756	128
		*Magnoliidae*	*Cinnamomum micranthum*	RWR91461.1	A0A443PKZ8	No	33–156	513	3	3	Far	22.7	2.044	112
ricin-B containing protein	SAR	*Stramenopiles*	*Achlya hypogyna*	AIG56170.1	A0A0A7CNX2	Yes	624–745	670	2	2	*a*			
		*Stramenopiles*	*Saprolegnia diclina*	EQC42503.1	T0SAX9	No	342–461	301	2	2	Close	49.6	0.884	64
		*Stramenopiles*	*Thraustotheca clavata*	OQR95700.1	A0A1V9ZCY1	No	235–351	275	2	2	Far	45.2	1.564	64

From each example cluster, the AlphaFold model of one CBM13, one closely and one remotely related CBM13-predicted ricin-B lectin module was selected and structurally compared. *a*: highlights the CBM13 modules itself, so no phylogenetical relatedness or structural comparison to itself can be calculated

## Conclusions

This study focussed on several aspects related to the classification of the CBM13 modules and ricin-B lectin domains. Findings from this study are of particular importance for the glycobiology community. The presented ricin-B/CBM13 study is exemplary for cases where there is overlap between the classification of lectins and CBMs. Furthermore, this study demonstrates how SSNs can be utilised to study the sequence-structure-function relationships, resulting in the identification of putative ricin-B lectins with close structural resemblance towards CBM13. Therefore, the CBM13 family may be larger than initially expected. This study is limited to predictions and simulations. Therefore, several of the drawn conclusions are awaiting biological validation.

We first demonstrated that CBM13 proteins and putative ricin-B lectins make use of the same protein domain identifiers, rendering it difficult to distinguish them based on semantics and nomenclature use. We established that CBM13 entries are usually attributed with domain identifiers from more databases compared to putative ricin-B lectin entries, and that only a minority of the CBM13 entries present in UniProt are equipped with the CAZy cross-reference. Therefore, it could be considered to merge these groups of proteins, although sufficient counterarguments exist to maintain putative ricin-B lectins and CBM13 proteins as separate groups. We elaborated on suggestions to guard the distinction between CAZy and non-CAZy entries in the public databases, since several issues were identified. Extension and curation of the existing inter-database connection between UniProt and CAZy could resolve these issues. This study, dealing with problems arising from overlap between ricin-B lectin classification and CBM13 classification can be extended to other cases. Therefore, we also suggest the introduction of novel CBM identifiers in UniProt and InterPro.

Ricin-B lectins and CBM13 proteins are diverse groups of associated proteins, fulfilling a plethora of functions in all taxonomical lineages. We investigated the occurrence and distribution of proteins with CAZyme activity, lectin activity and other activities based on the assigned protein name and GO terms but found no significant differences between both groups. However, it is very striking that the large majority of putative ricin-B lectins are characterised by names actively referring to ricin-B lectins, although GO terms point in a completely different direction in terms of proposed functionality. Moreover, making use of SSNs, we showed that many predicted ricin-B lectins from different taxonomical origins are attributed with protein names and GO terms referring to CAZyme activity. Furthermore, we demonstrated that many CBM13-predicted ricin-B lectin modules are highly similar to CBM13 modules, based on sequence conservation and structural resemblances, and are therefore interesting candidates to investigate CBM13 membership, particularly in view of the protein structure-function relationships [96]. Our investigations exemplify that CBMs and the carbohydrate-binding domain of lectins overlap in nomenclature, structure and function.

Traditionally, the biological function of a protein is often inferred by means of sequence homology, assuming that similar sequences share a similar structure and therefore also a similar function [97]. However, protein structure is more conserved than its primary sequence, meaning that proteins with similar structure (and therefore function) may originate from primary sequences that not necessarily show high similarity [96]. Because of the low mutual sequence homology, most putative ricin-B lectin entries are not part of the CBM13 family. Nevertheless, we demonstrated that several important repeats and residues (i.e. QXW and cysteines) are conserved amongst CBM13 and ricin-B lectin domains. Despite the conserved residues, repeats and similar structure, it remains unsure whether putative ricin-B lectin modules with similar structure to the characterised CBM13 modules will exhibit the same function. Additionally, performing modelling and molecular dynamics could be considered to unravel putative structural and mechanistical properties. Therefore, it must be stressed that biochemical characterisation remains of paramount importance to validate the *in silico* predictions originating from this study. However, it could be considered that putative ricin-B lectins and CBM13s are distant members of a larger ricin-B/CBM13 superfamily, displaying distinctive carbohydrate-binding properties.

### Electronic supplementary material

Below is the link to the electronic supplementary material.

Supplementary Material 1

Supplementary Material 2

Supplementary Material 3

Supplementary Material 4

Supplementary Material 5

Supplementary Material 6

Supplementary Material 7

Supplementary Material 8

Supplementary Material 9

Supplementary Material 10

Supplementary Material 11

Supplementary Material 12

Supplementary Material 13

Supplementary Material 14

Supplementary Material 15

## Data Availability

The datasets supporting the conclusions of this article are included within the article and its supplementary files.

## Abbreviations

AA: Auxiliary activities
CAZy: Carbohydrate-active enzymes database
CBM: Carbohydrate-binding modules
CE: Carbohydrate esterases
df: Degrees of freedom
GH: Glycoside hydrolases
GO: Gene ontology
GT: Glycosyl transferases
PE: Protein existence
PL: Polysaccharide lyases
RIP: Ribosome-inactivating protein
RMSD: Root-mean-square deviation
SAR: Stramenopiles-Alveolata-Rhizaria
SSN: Sequence similarity network
