# Supplementary material for: Investigating diversity and similarity between CBM13 modules and ricin-B lectin domains using sequence similarity networks
Source: BMC Genomics. 2024 Jun 27;25:643. doi: 10.1186/s12864-024-10554-1 (PMC11212257; doi:10.1186/s12864-024-10554-1)
Supplement: Supplementary file 7 — Supplementary Material 7 [file 12864_2024_10554_MOESM7_ESM.docx]

**Supplementary File S7: topology of the SSN at different E-value thresholds.** Below, different topologies of the SSN at different E-value thresholds are shown, ranging from 10^-20^ till 10^-100^.

| **SSN at threshold 10^-20^** |
| --- |
| 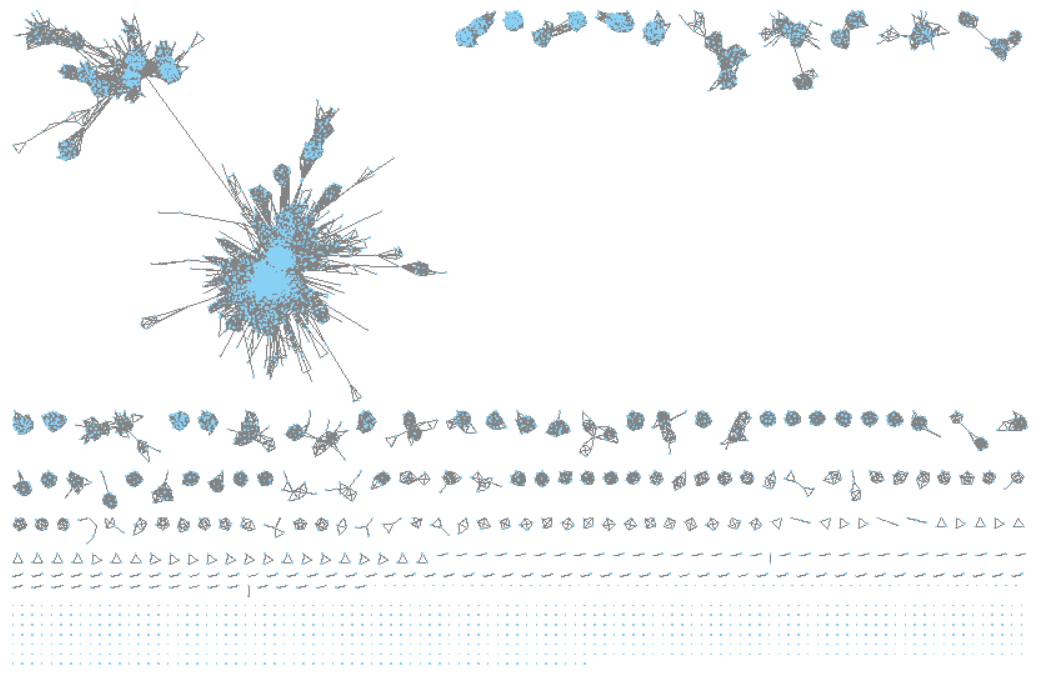 |

| **SSN at threshold 10^-30^** |
| --- |
| 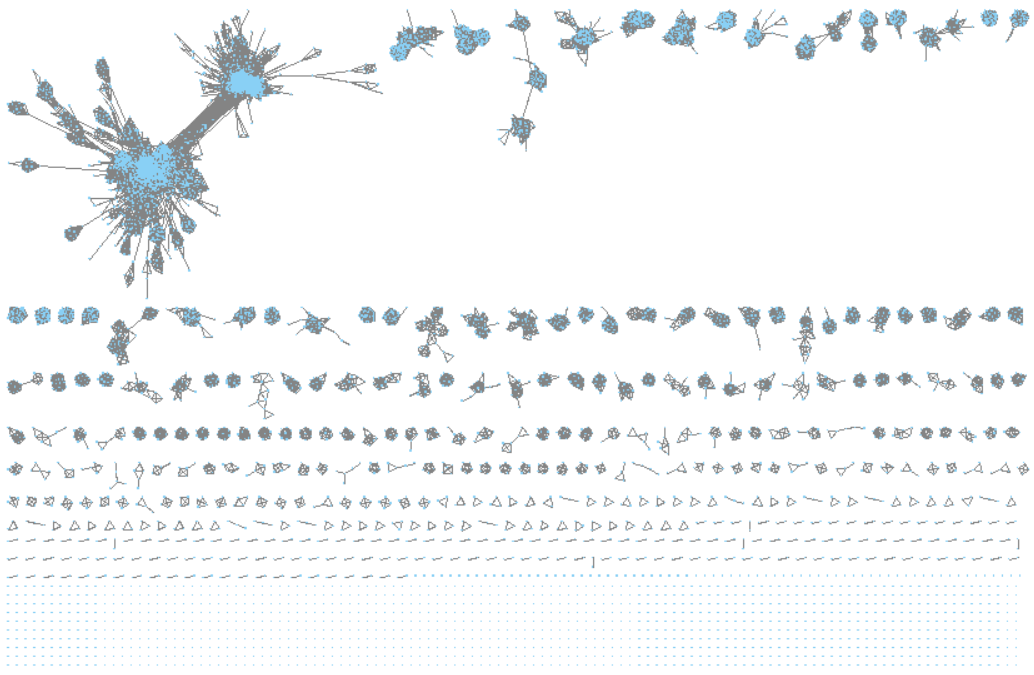 |

| **SSN at threshold 10^-40^** |
| --- |
| 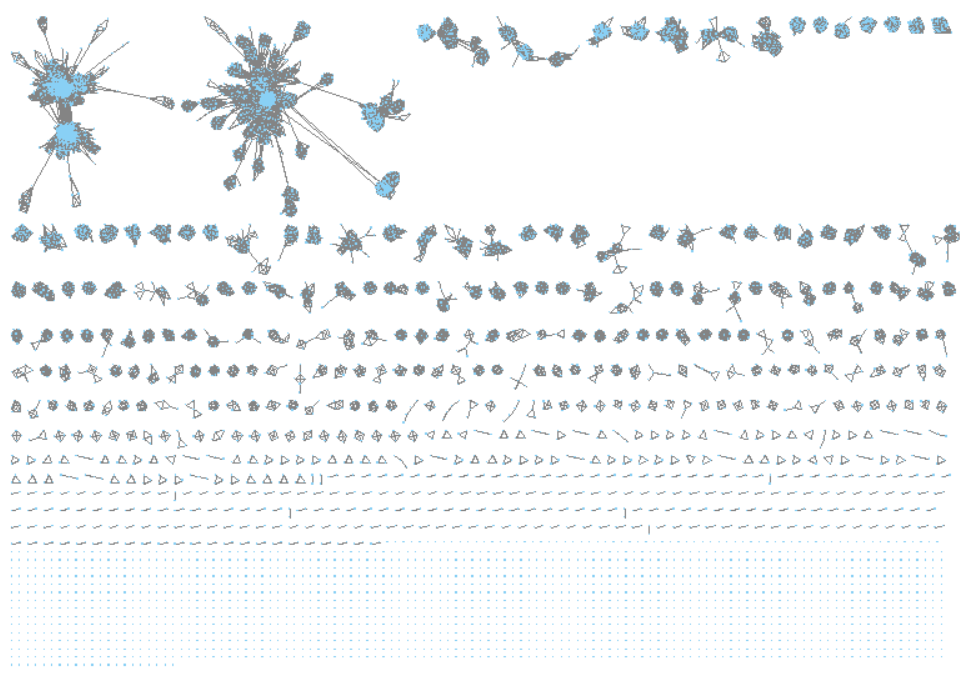 |

| **SSN at threshold 10^-100^** |
| --- |
| 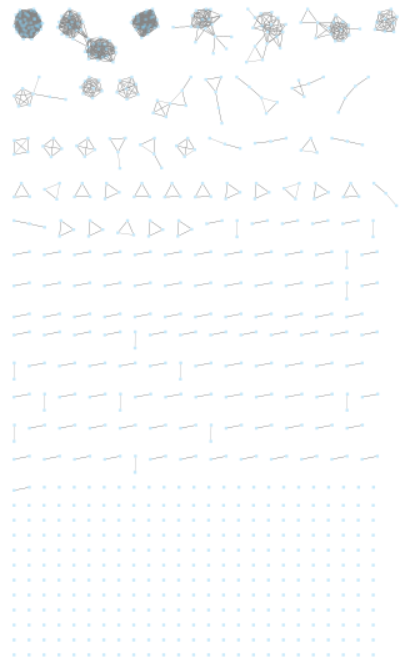 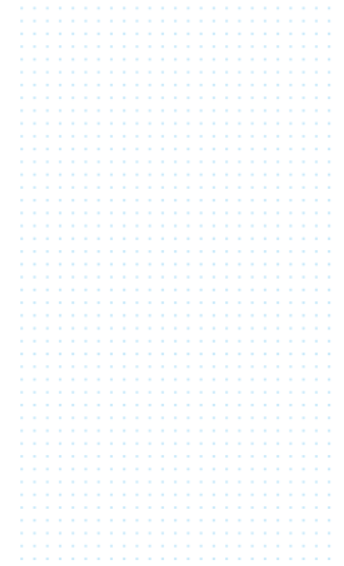 |
