# Supplementary material for: Investigating diversity and similarity between CBM13 modules and ricin-B lectin domains using sequence similarity networks
Source: BMC Genomics. 2024 Jun 27;25:643. doi: 10.1186/s12864-024-10554-1 (PMC11212257; doi:10.1186/s12864-024-10554-1)
Supplement: Supplementary file 9 — Supplementary Material 9 [file 12864_2024_10554_MOESM9_ESM.docx]

**Supplementary File S9A: Comparison of BLAST-predicted and InterPro-predefined module lengths and presence of QX[F;W;Y] motifs.**

| **Genbank ID** | **Module coordinates (BLAST)** | | **Module coordinates (InterPro)** | | **Length (BLAST)** | **Length (InterPro)** | **Length difference** | **QX[F;W;Y] motifs outside of predicted module** | **Type of QX[FWY] motif** | **BLAST score** |
| --- | --- | --- | --- | --- | --- | --- | --- | --- | --- | --- |
|  | *Start* | *End* | *Start* | *End* |  |  |  |  |  |  |
| AAA32625 | 274 | 401 | 272 | 399 | 128 | 128 | 0 | 0 | - | 675 |
| ACU38183 | 38 | 165 | 33 | 162 | 128 | 130 | 2 | 0 | - | 675 |
| BAA36393 | 129 | 260 | 131 | 258 | 132 | 128 | -4 | 0 | - | 695 |
| BAZ32339 | 9 | 138 | 3 | 136 | 130 | 134 | 4 | 0 | - | 242 |
| CAG2249197 | 557 | 680 | 559 | 679 | 124 | 121 | -3 | 0 | - | 196 |
| EAQ67128 | 586 | 684 | 561 | 694 | 99 | 109 | 35 | 1 | QKF | 156 |
| GEK00516 | 383 | 513 | 382 | 512 | 131 | 131 | 0 | 0 | - | 542 |
| HCZ74701 | 427 | 557 | 428 | 554 | 131 | 127 | -4 | 0 | - | 656 |
| JAV29930 | 373 | 491 | 376 | 484 | 119 | 109 | -10 | 0 | - | 233 |
| KOX40828 | 535 | 677 | 537 | 672 | 143 | 136 | -7 | 0 | - | 649 |
| LAB60325 | 215 | 345 | 222 | 340 | 131 | 119 | -12 | 0 | - | 683 |
| MUN35854 | 53 | 167 | 40 | 168 | 115 | 129 | 14 | 0 | - | 639 |
| NII05187 | 362 | 463 | 358 | 445 | 102 | 88 | -14 | 0 | - | 105 |
| OAM16885 | 74 | 184 | 65 | 185 | 111 | 121 | 10 | 0 | - | 163 |
| PKF81495 | 265 | 405 | 267 | 410 | 141 | 144 | 3 | 0 | - | 108 |
| QES47169 | 121 | 244 | 118 | 246 | 124 | 129 | 5 | 0 | - | 284 |
| RQX12202 | 115 | 204 | 121 | 208 | 90 | 88 | -2 | 0 | - | 103 |
| SBV24963 | 768 | 896 | 755 | 895 | 129 | 141 | 12 | 0 | - | 589 |
| TCJ39650 | 292 | 414 | 278 | 414 | 123 | 137 | 14 | 0 | - | 535 |
| VDB90017 | 66 | 171 | 38 | 172 | 106 | 135 | 29 | 0 | - | 104 |

**Supplementary File S9B: Screenshot of the InterPro webpage of genbank ID EAQ67128.** The red rectangle indicates the predefined module. Module coordinates are extracted by hovering over the highlighted module (indicated with the red box).


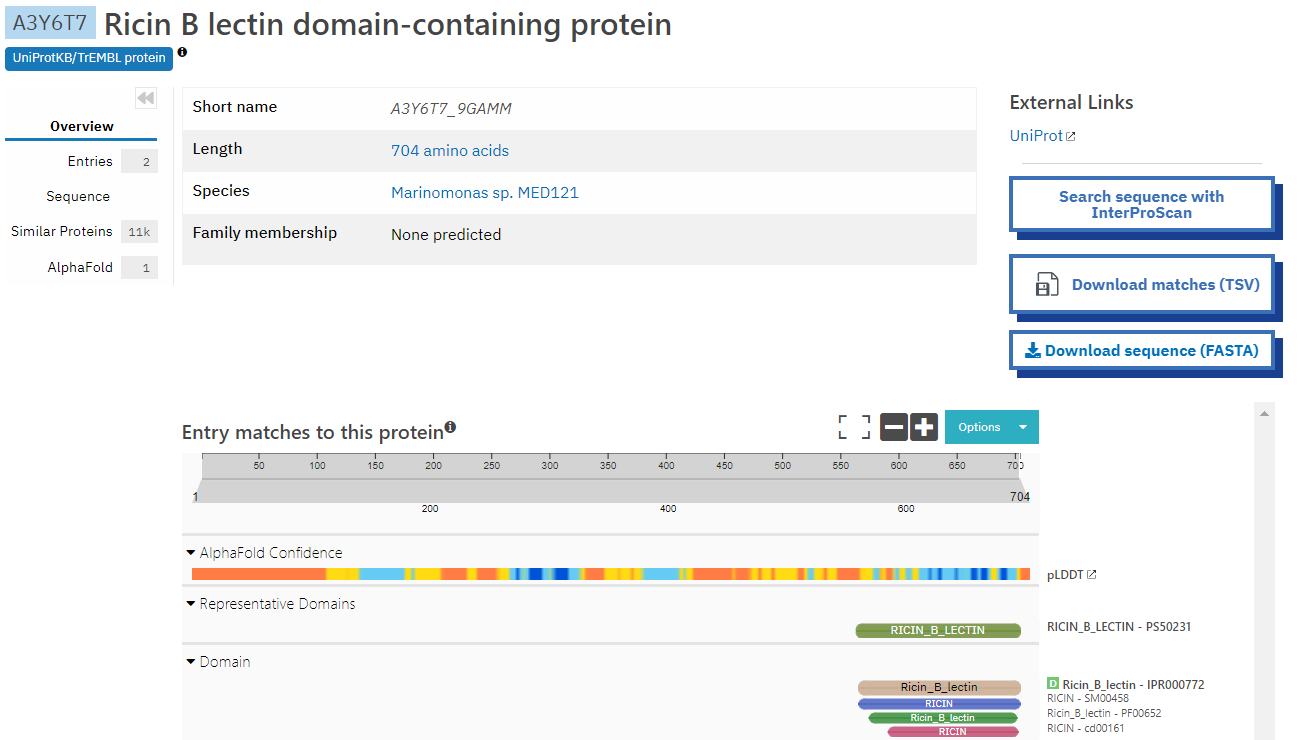


**Supplementary File S9C: Multiple sequence alignment of the CBM13-predicted ricin-B lectin module and its full length amino acid sequence.** The CBM13-predicted ricin-B lectin module is denoted with “mod”, while the full-length amino acid sequence is denoted with “full”. Yellow: predefined module length, given by InterPro. Cyan: predicted module length. **Bold and red**: QX[F;W;Y] motif found adjacent to the predicted module but part of the module as defined by InterPro.

EAQ67128.1_586-684mod ------------------------------------------------------------ 0

EAQ67128.1full MKKTLLYLSMLASASSVYADTGDQCSAYIDRVESNGGVTYNIESAAILSANTAITTGVEV 60

EAQ67128.1_586-684mod ------------------------------------------------------------ 0

EAQ67128.1full LGQSTLSAVFPYASIILRQITPGETTAAADPDLVACLESFDSRLTTLETYLLAEEASIAL 120

EAQ67128.1_586-684mod ------------------------------------------------------------ 0

EAQ67128.1full NAVRSKIQKDIIEQHDAVANFDVYESYDVIAAKLAGTLSYFLHEDQIENADADIYETIKN 180

EAQ67128.1_586-684mod ------------------------------------------------------------ 0

EAQ67128.1full DTLLYLLSAQNSAFLHNLEFNAYCAKEKNYQWTSTATSYIEHNDSKNHALMDYDLLKSLS 240

EAQ67128.1_586-684mod ------------------------------------------------------------ 0

EAQ67128.1full DFGYSTEDLQESNFDITDCELGRDSHSRYKALLSENTSYSPIAQIYDDLGFDITLDSGNS 300

EAQ67128.1_586-684mod ------------------------------------------------------------ 0

EAQ67128.1full NYLDVAIDIDSSKLADYRKGLIGSCSHSVDWGIAGHSGRDPWGTLILGGAKITTTSKDYG 360

EAQ67128.1_586-684mod ------------------------------------------------------------ 0

EAQ67128.1full GTATFSKSVIAGRNKIDSAKRYNEGYCDTWVGNLEDDLDDEVITLLNAIKVSLKNLSMYQ 420

EAQ67128.1_586-684mod ------------------------------------------------------------ 0

EAQ67128.1full LVRPAYELAGRAPQFLTYETGVIDNDLLESYMLVPQFDVNDEGDISDNYNHYQLFNAANG 480

EAQ67128.1_586-684mod ------------------------------------------------------------ 0

EAQ67128.1full DRYLKHRFDVGIVARNNGSGIDSHWQLSQRWPGEKTRMMNVYTNECVVISDAGELHMRSC 540

EAQ67128.1_586-684mod ---------------------------------------------SNSNKIAATDCDITN 15

EAQ67128.1full DEYPIAYVWDIHANTSGAVDASLKLSSNSHKNQCVLVSDETMPEYSNSNKIAATDCDITN 600

***************

EAQ67128.1_586-684mod ANQTFVLGDNNDIRVNGICLDVPRSEAFAGQSVIVYSCHYGDNQSWDLNPDGTIQSALNA 75

EAQ67128.1full ANQTFVLGDNNDIRVNGICLDVPRSEAFAGQSVIVYSCHYGDNQSWDLNPDGTIQSALNA 660

************************************************************

EAQ67128.1_586-684mod VDGTNLCLEANDATSDIAITLQAC-------------------- 99

EAQ67128.1full VDGTNLCLEANDATSDIAITLQACDSSNPA**QKF**IPNEIVEEEEV 704

************************
