# Supplementary material for: Investigating diversity and similarity between CBM13 modules and ricin-B lectin domains using sequence similarity networks
Source: BMC Genomics. 2024 Jun 27;25:643. doi: 10.1186/s12864-024-10554-1 (PMC11212257; doi:10.1186/s12864-024-10554-1)
Supplement: Supplementary file 10 — Supplementary Material 10 [file 12864_2024_10554_MOESM10_ESM.docx]

**Supplementary File S10**: **Comparison and ranking of Gene Ontology occurring within the CBM13 and putative ricin-B lectin SSN subdivisions.**

| **Rank** | **CBM13** | | **Putative ricin-B lectins** | |
| --- | --- | --- | --- | --- |
|  | **GO term** | **Hits** | **GO term** | **Hits** |
| 1 | Carbohydrate binding [GO:0030246] | 234 | Carbohydrate binding [GO:0030246] | 2405 |
| 2 | Hydrolase activity, hydrolyzing O-glycosyl compounds [GO:0004553] | 197 | Hydrolase activity, hydrolyzing O-glycosyl compounds [GO:0004553] | 901 |
| 3 | α-L-arabinofuranosidase activity [GO:0046556] | 70 | Glycosyltransferase activity [GO:0016757] | 589 |
| 4 | Hydrolase activity [GO:0016787] | 58 | Polypeptide N-acetylgalactosaminyltransferase activity [GO:0004653] | 582 |
| 5 | Endo-1,4-β-xylanase activity [GO:0031176] | 49 | Hydrolase activity [GO:0016787] | 276 |
| 6 | Raffinose α-galactosidase activity [GO:0052692] | 38 | α-L-arabinofuranosidase activity [GO:0046556] | 266 |
| 7 | Glycosyltransferase activity [GO:0016757] | 29 | Raffinose α-galactosidase activity [GO:0052692] | 170 |
| 8 | Glucosylceramidase activity [GO:0004348] | 29 | Metal ion binding [GO:0046872] | 157 |
| 9 | Polypeptide N-acetylgalactosaminyltransferase activity [GO:0004653] | 26 | Transferase activity [GO:0016740] | 146 |
| 10 | Serine-type endopeptidase activity [GO:0004252] | 21 | Endo-1,4-β-xylanase activity [GO:0031176] | 137 |
| 11 | Pectate lyase activity [GO:0030570] | 19 | Glucosylceramidase activity [GO:0004348] | 128 |
| 12 | Hydrolase activity, acting on ester bonds [GO:0016788] | 16 | Hydrolase activity, acting on glycosyl bonds [GO:0016798] | 74 |
| 13 | Toxin activity [GO:0090729] | 14 | Feruloyl esterase activity [GO:0030600] | 69 |
| 14 | Feruloyl esterase activity [GO:0030600] | 12 | ATP binding [GO:0005524] | 58 |
| 15 | Transferase activity [GO:0016740] | 12 | Pectate lyase activity [GO:0030570] | 57 |
| 16 | Chitin binding [GO:0008061] | 11 | Hydrolase activity, acting on ester bonds [GO:0016788] | 57 |
| 17 | rRNA N-glycosylase activity [GO:0030598] | 11 | Metalloendopeptidase activity [GO:0004222] | 48 |
| 18 | Metal ion binding [GO:0046872] | 10 | Serine-type endopeptidase activity [GO:0004252] | 48 |
| 19 | Galactosylceramidase activity [GO:0004336] | 10 | Toxin activity [GO:0090729] | 46 |
| 20 | α-L-fucosidase activity [GO:0004560] | 9 | Galactosylceramidase activity [GO:0004336] | 45 |
|  | | | | |
