# Supplementary material for: Investigating diversity and similarity between CBM13 modules and ricin-B lectin domains using sequence similarity networks
Source: BMC Genomics. 2024 Jun 27;25:643. doi: 10.1186/s12864-024-10554-1 (PMC11212257; doi:10.1186/s12864-024-10554-1)
Supplement: Supplementary file 13 — Supplementary Material 13 [file 12864_2024_10554_MOESM13_ESM.docx]

**Supplementary File S13: WebLogos illustrating sequence conservation amongst CBM13 modules and ricin-B lectin domains.** WebLogos were generated by using the CBM13 module sequences and the predicted ricin-B lectin domain sequences from the representative clusters of *Metazoa*, *Bacteria*, *Fungi*, *Viridiplantae* and SAR.

| **A: *Metazoa*** |
| --- |
| 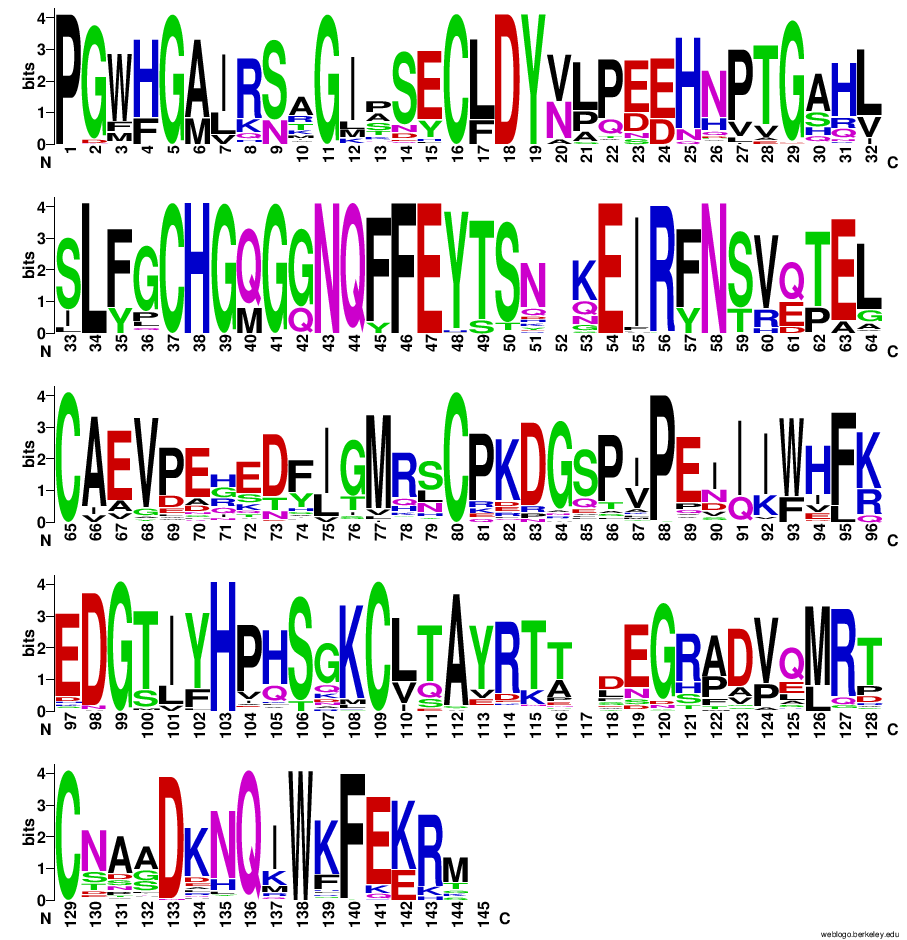 |

| **B: *Bacteria*** |
| --- |
| 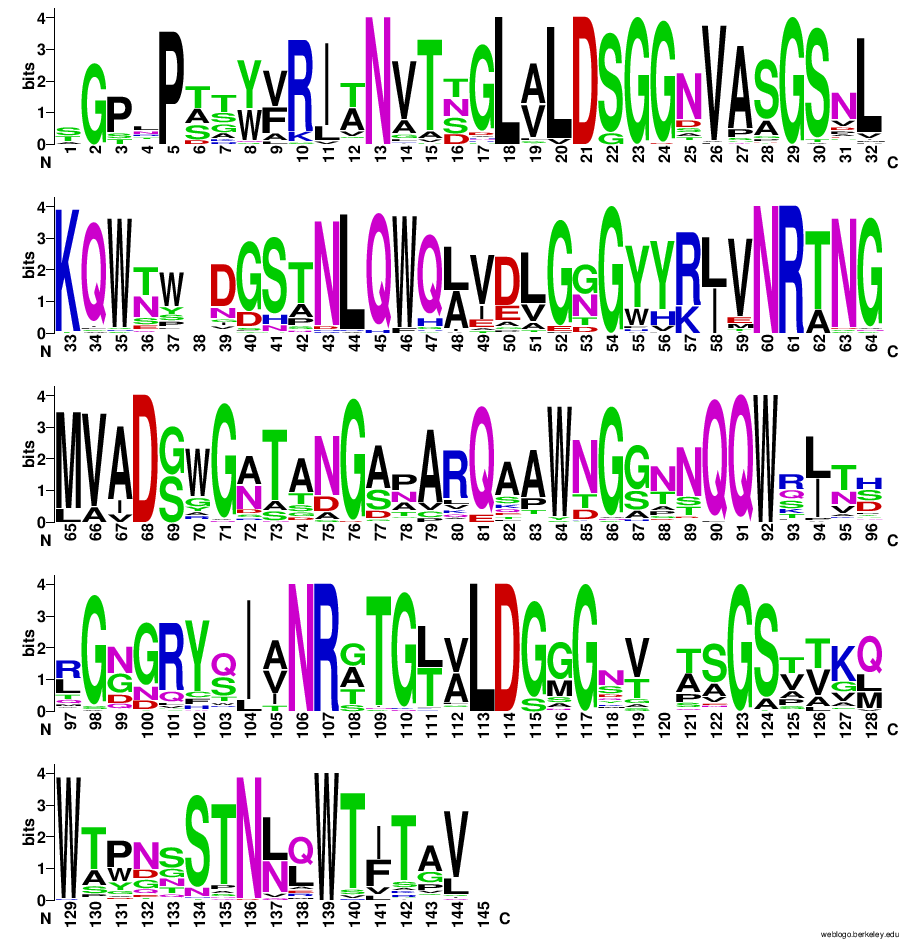 |

| **C: *Fungi*** |
| --- |
| 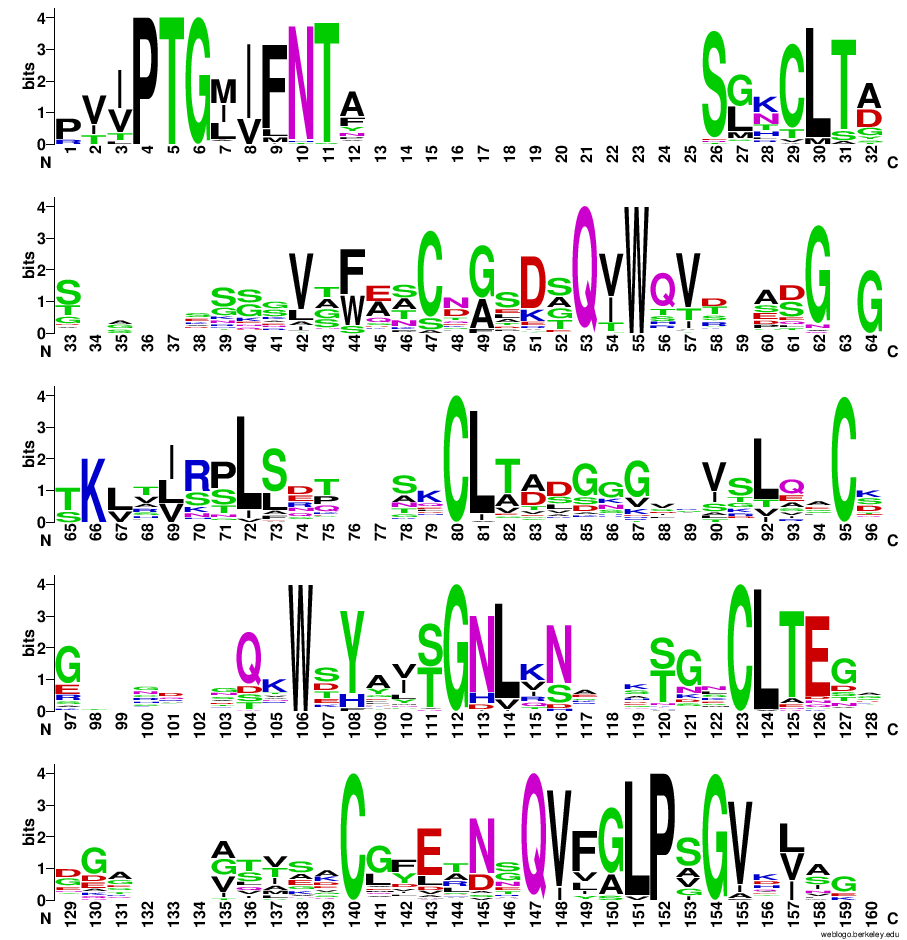 |

| **D: *Viridiplantae*** |
| --- |
| 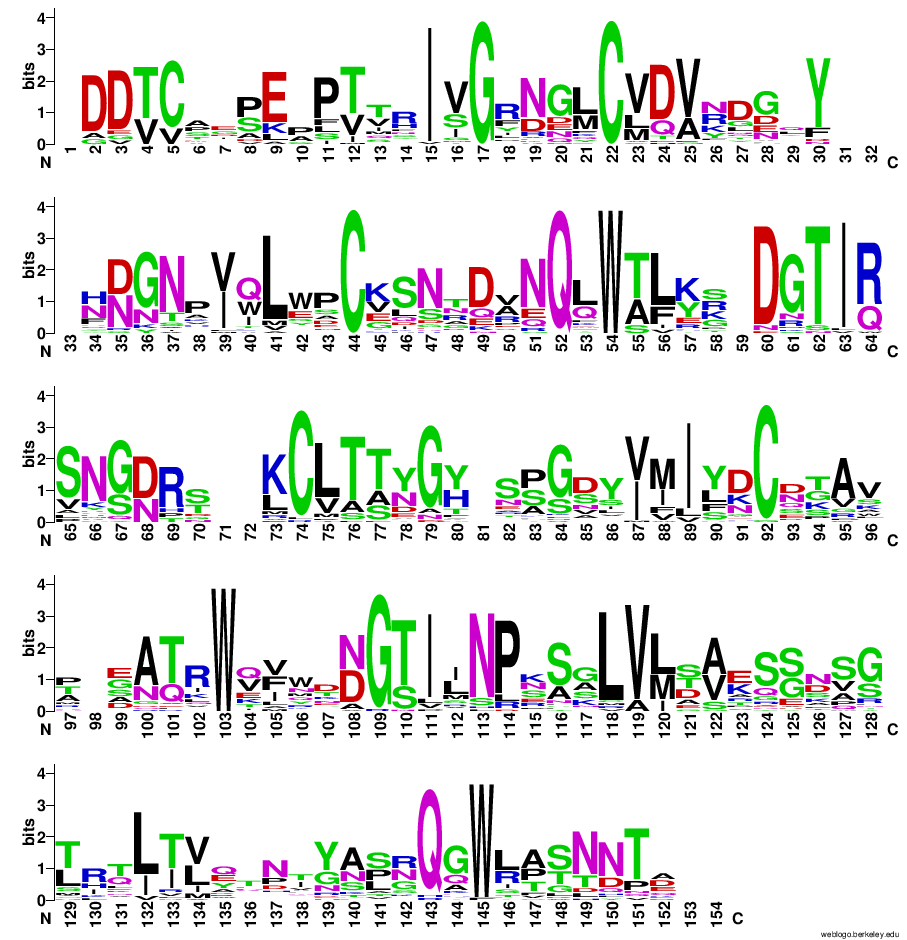 |

| **E: SAR** |
| --- |
| 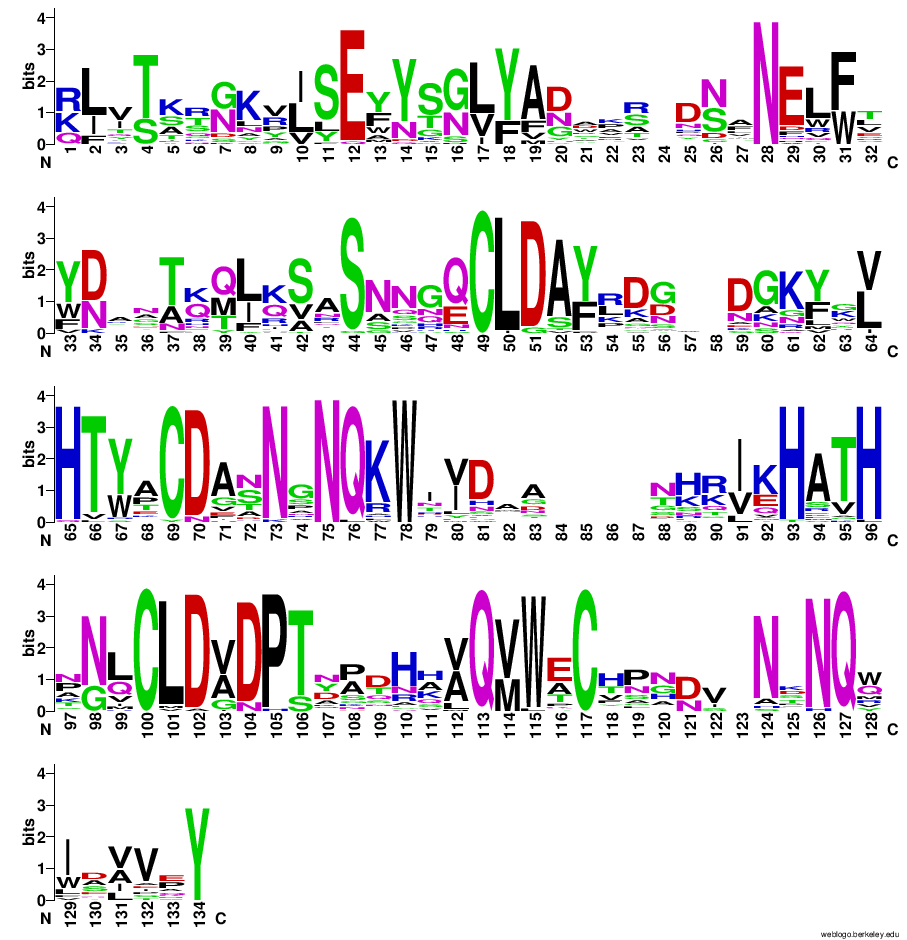 |
