# Supplementary material for: Investigating diversity and similarity between CBM13 modules and ricin-B lectin domains using sequence similarity networks
Source: BMC Genomics. 2024 Jun 27;25:643. doi: 10.1186/s12864-024-10554-1 (PMC11212257; doi:10.1186/s12864-024-10554-1)
Supplement: Supplementary file 14 — Supplementary Material 14 [file 12864_2024_10554_MOESM14_ESM.docx]

**Supplementary File S13: Phylogenetic trees depicting the evolutionary distance between CAZy-CBM13 and putative ricin-B lectin modules.** Representative clusters of modules were manually selected from the ricin-B/CBM13 SSN at threshold level E = 10^-30^. Modules were submitted to the phylogeny.fr pipeline to create phylogenetical trees. Bootstrap values are shown on the branches and are expressed as percentages. The scale bar indicates the number of substitutions per site according to a maximum likelihood estimation. Each branch tip represents a CBM13-predicted ricin-B lectin or CAZy-CBM13 module from the chosen SSN cluster. Accessions highlighted in red are CAZy-CBM13 accessions.

| **A: *Metazoa*** |
| --- |
| 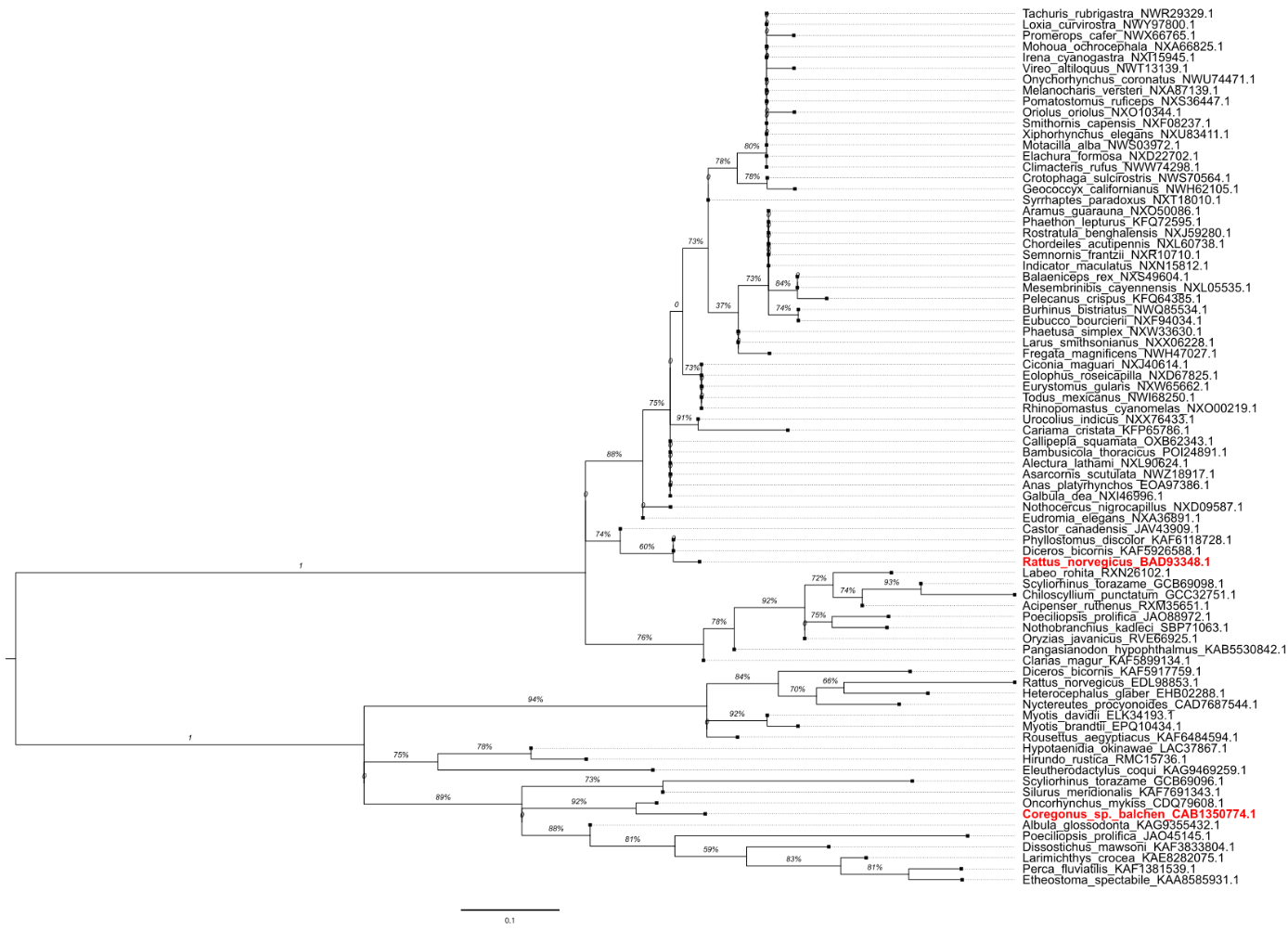 |

| **B: *Bacteria*** |
| --- |
| 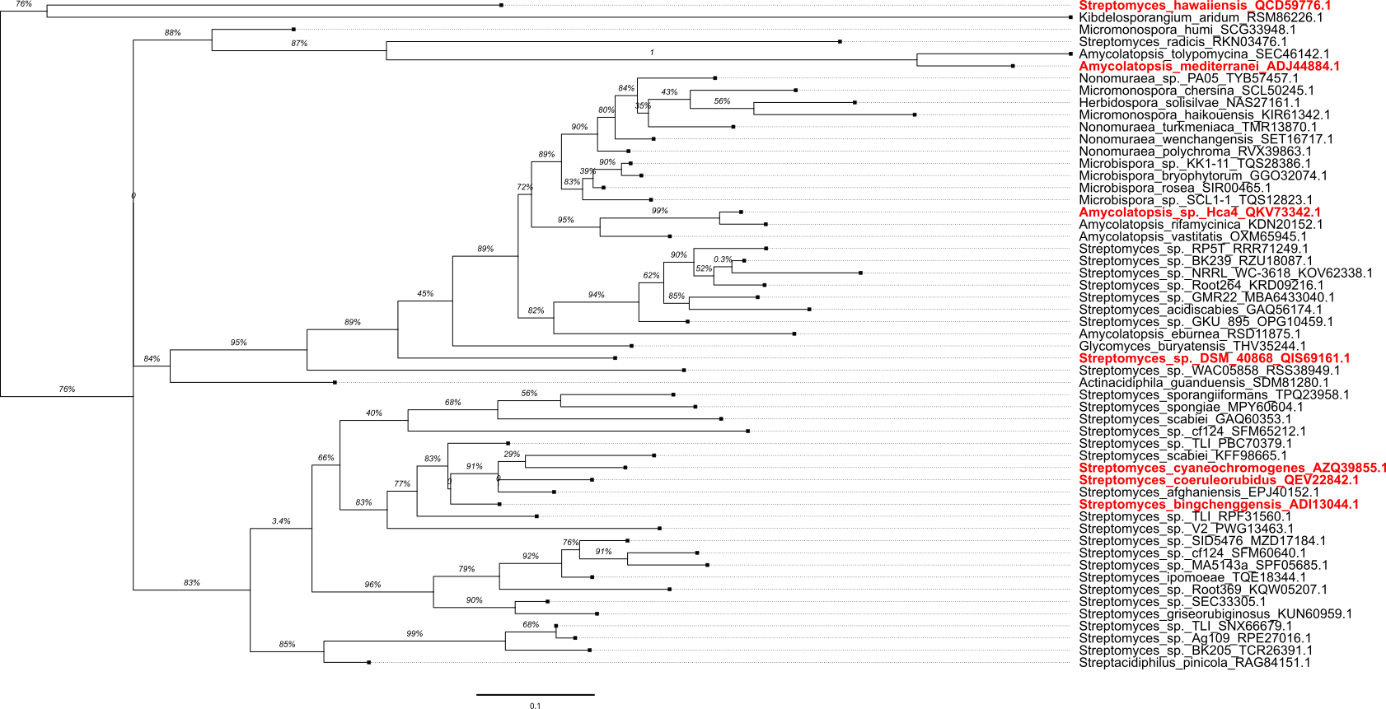 |

| **C: *Fungi*** |
| --- |
| 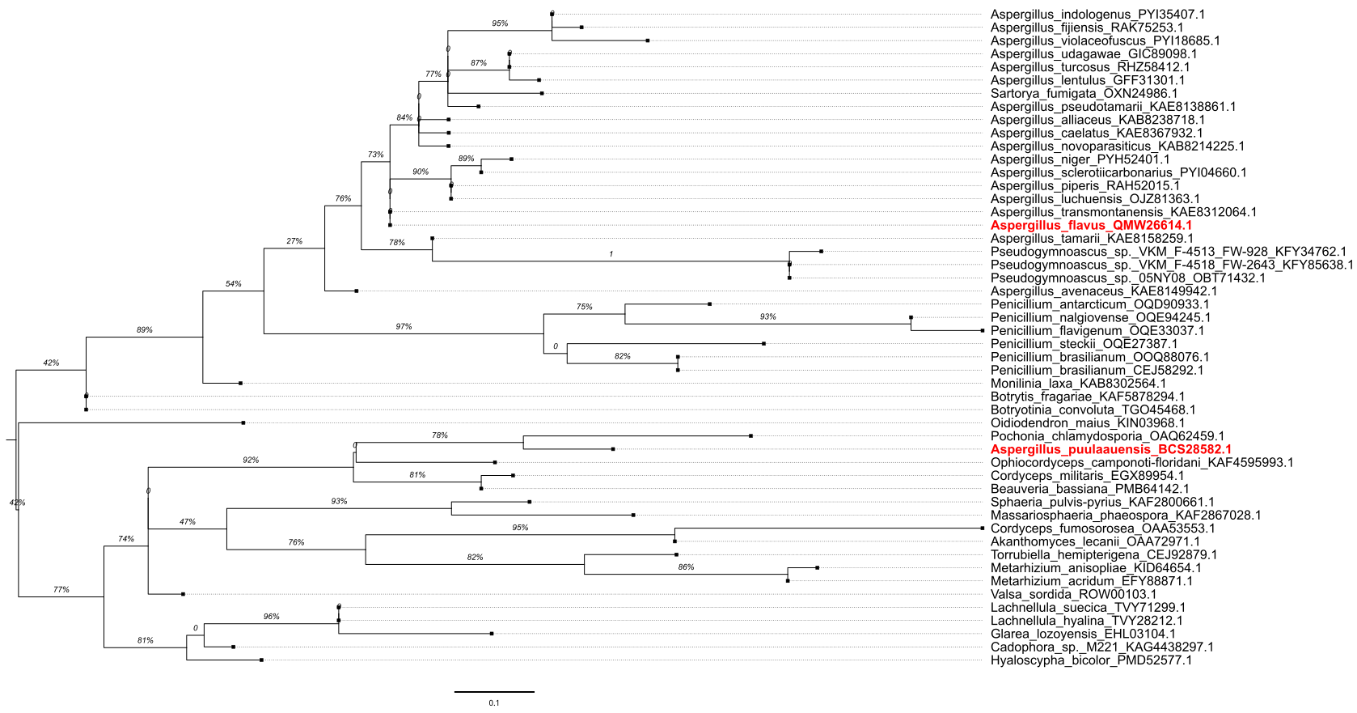 |

| **D: *Viridiplantae*** |
| --- |
| 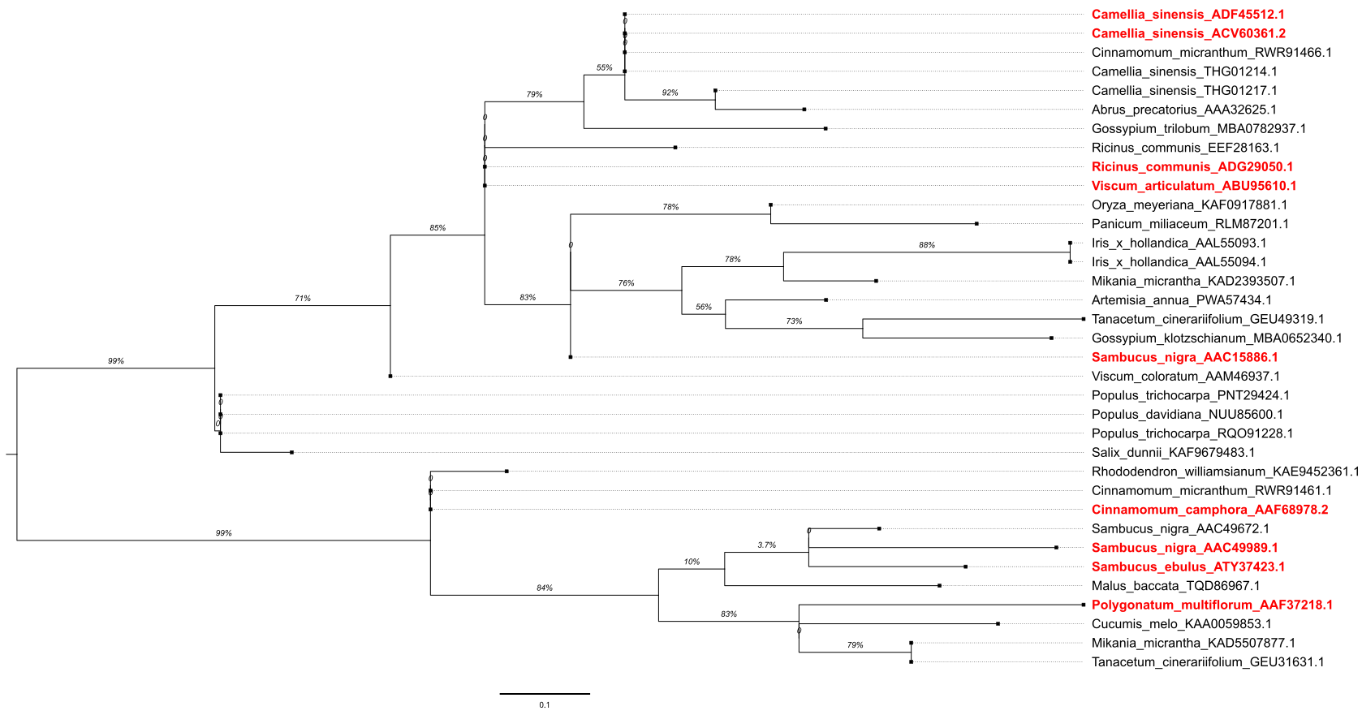 |

| **E: SAR** |
| --- |
| 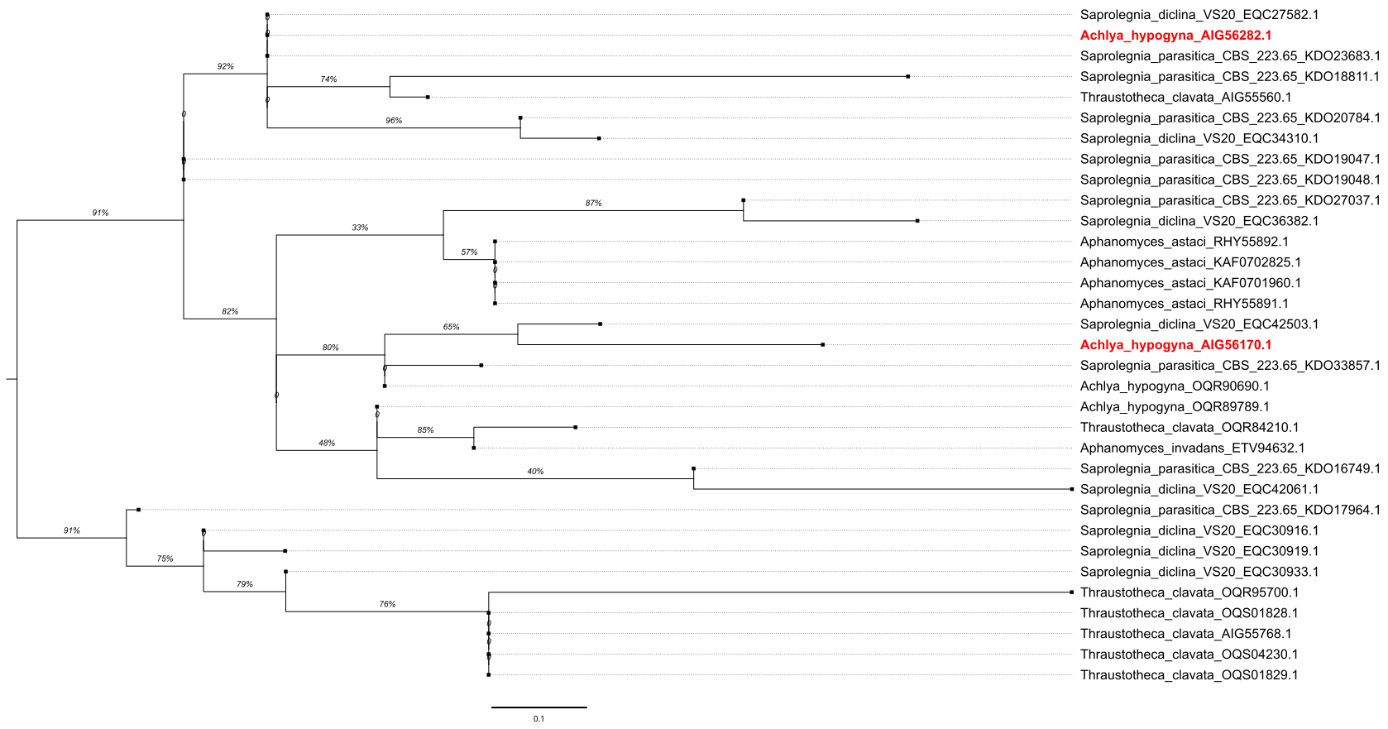 |
