# Supplementary material for: Investigating diversity and similarity between CBM13 modules and ricin-B lectin domains using sequence similarity networks
Source: BMC Genomics. 2024 Jun 27;25:643. doi: 10.1186/s12864-024-10554-1 (PMC11212257; doi:10.1186/s12864-024-10554-1)
Supplement: Supplementary file 15 — Supplementary Material 15 [file 12864_2024_10554_MOESM15_ESM.docx]

**Supplementary file S0: overview and description of all supplementary files of this study.**

| **File name** | **File type + size** | **Description** |
| --- | --- | --- |
| S1 sequence space_part1.xlsx | Excel worksheet  2355 kB | Part 1 of the ricin-B/CBM13 sequence space containing the n = 6521 CBM13 entries. |
| S2 sequence space_part2.xlsx | Excel worksheet  13686 kB | Part 2 of the ricin-B/CBM13 sequence space containing the n = 44283 putative ricin-B lectin entries, with Genbank IDs starting from A till M. |
| S3 sequence space_part3.xlsx | Excel worksheet  12751 kB | Part 3 of the ricin-B/CBM13 sequence space containing the n = 40263 putative ricin-B lectin entries, with Genbank IDs starting from N till Z. |
| S4 CAZy cross-references.xlsx | Excel worksheet  26 kB | Overview of entries featured with a CBM13 cross-reference in UniProt. |
| S5 SSN network file.txt | txt file  55292 kB | Network file of the SSN containing all pairwise comparisons generated by all-vs-all BLAST at threshold E level 10^-30^. |
| S6 SSN nodes list.txt | Excel worksheet  1739 kB | Nodes list containing the biological metadata of the complete SSN. |
| S7 SSN topologies.docx | Word document  681 kB | Images of the topologies of the SSNs at different threshold E values. |
| S8 SSN nodes list at E = 10-30 | Excel worksheet  1654 kB | Nodes list containing the biological metadata of the SSN at E-value threshold level 10-30. |
| S9 module length and QX[F;W;Y] modules.docx | Word document  105 kB | 9A: Comparison of module length and number of QX[F;Y;W] motifs in predicted ricin-B lectin motifs compared to InterPro-predefined modules. 9B: Screenshot of the InterPro webpage of Genbank ID EAQ67128. 9C: Multiple sequence alignment of the CBM13-predicted ricin-B lectin module and its full-length amino acid sequence. |
| S10 comparison of GO terms.docx | Word document  16 kB | Comparison and ranking of Gene Ontology terms that occur within the CBM13 and putative ricin-B lectin SSN subdivisions. |
| S11 protein names and GO terms.xlsx | Excel worksheet  501 kB | Overview of protein names and GO terms belonging to entries from the CBM13 and putative ricin-B lectin SSN subdivisions.  Sheet 1: overview of the complete SSN; Sheet 2: overview of all putative ricin-B lectin entries with CAZyme name; Sheet 3: overview of all putative ricin-B lectin entries with CAZyme GO terms |
| S12 Example clusters.xlsx | Excel worksheet  35 kB | Overview of selected CBM13-predicted ricin-B lectin and CBM13 modules from different taxonomical origins: *Metazoa* (red), *Bacteria* (blue), *Fungi* (pink), *Viridiplantae* (orange) and SAR (green). |
| S13 WebLogos illustrating sequence conservation.docx | Word document  555 kB | WebLogos illustrating sequence conservation amongst CBM13 modules and ricin-B lectin domains from different taxonomical origins. Subfiles: A (*Metazoa*), B (*Bacteria*), C (*Fungi*), D (*Viridiplantae*), E (SAR). |
| S14 Phylogenetic trees example clusters.docx | Word document  1060 kB | Phylogenetic trees depicting the evolutionary distance between CBM13 and CBM13-predicted ricin-B lectin modules from different taxonomical origins. Subfiles: A (*Metazoa*), B (*Bacteria*), C (*Fungi*), D (*Viridiplantae*), E (SAR). |
